# The Cardioprotective Effects of Semaglutide Exceed Those of Dietary Weight Loss in Mice With HFpEF

**DOI:** 10.1016/j.jacbts.2023.05.012

**Published:** 2023-07-26

**Authors:** Coenraad Withaar, Laura M.G. Meems, Edgar E. Nollet, E. Marloes Schouten, Marie A. Schroeder, Lotte B. Knudsen, Kristoffer Niss, Christian T. Madsen, Annabelle Hoegl, Gianluca Mazzoni, Jolanda van der Velden, Carolyn S.P. Lam, Herman H.W. Silljé, Rudolf A. de Boer

**Affiliations:** a University of Groningen, University Medical Center Groningen, Department of Cardiology, Groningen, the Netherlands; b Amsterdam University Medical Center, Vrije Universiteit Amsterdam, Physiology, Amsterdam, the Netherlands; c Amsterdam Cardiovascular Sciences, Heart Failure and Arrhythmias, Amsterdam, the Netherlands; d Research and Early Development, Novo Nordisk A/S, Bagsvaerd, Denmark; e Global Translation, Novo Nordisk A/S, Måløv, Denmar; f National Heart Centre Singapore & Duke-National University of Singapore, Singapore; g Department of Cardiology, Erasmus Medical Center, Rotterdam, the Netherlands

**Keywords:** cardiometabolic, diabetes, GLP-1RA, HFpEF, obesity, semaglutide

## Abstract

Obesity-related heart failure with preserved ejection fraction (HFpEF) has become a well-recognized HFpEF subphenotype. Targeting the unfavorable cardiometabolic profile may represent a rational treatment strategy. This study investigated semaglutide, a glucagon-like peptide-1 receptor agonist that induces significant weight loss in patients with obesity and/or type 2 diabetes mellitus and has been associated with improved cardiovascular outcomes. In a mouse model of HFpEF that was caused by advanced aging, female sex, obesity, and type 2 diabetes mellitus, semaglutide, compared with weight loss induced by pair feeding, improved the cardiometabolic profile, cardiac structure, and cardiac function. Mechanistically, transcriptomic, and proteomic analyses revealed that semaglutide improved left ventricular cytoskeleton function and endothelial function and restores protective immune responses in visceral adipose tissue. Strikingly, treatment with semaglutide induced a wide array of favorable cardiometabolic effects beyond the effect of weight loss by pair feeding. Glucagon-like peptide-1 receptor agonists may therefore represent an important novel therapeutic option for treatment of HFpEF, especially when obesity-related.

## Highlights

• Asks how the effects of semaglutide compare with the effects of weight loss by Pair Feeding (PF) in a mouse model of HFpEF and which cardioprotective effects of semaglutide are achieved in HFpEF.
• PF and semaglutide reduce bodyweight and adipose tissue to a similar degree.
• Semaglutide improves cardiometabolic profile, cardiac structure, and cardiac function.
• Semaglutide improves LV cytoskeleton function and endothelial function and restores protective immune responses in VAT.
• Semaglutide prompts a wide array of favorable cardiometabolic effects beyond that of weight loss by PF. Semaglutide may therefore represent an important novel therapeutic option for obesity-related HFpEF.

## Visual Abstract

**Figure:**
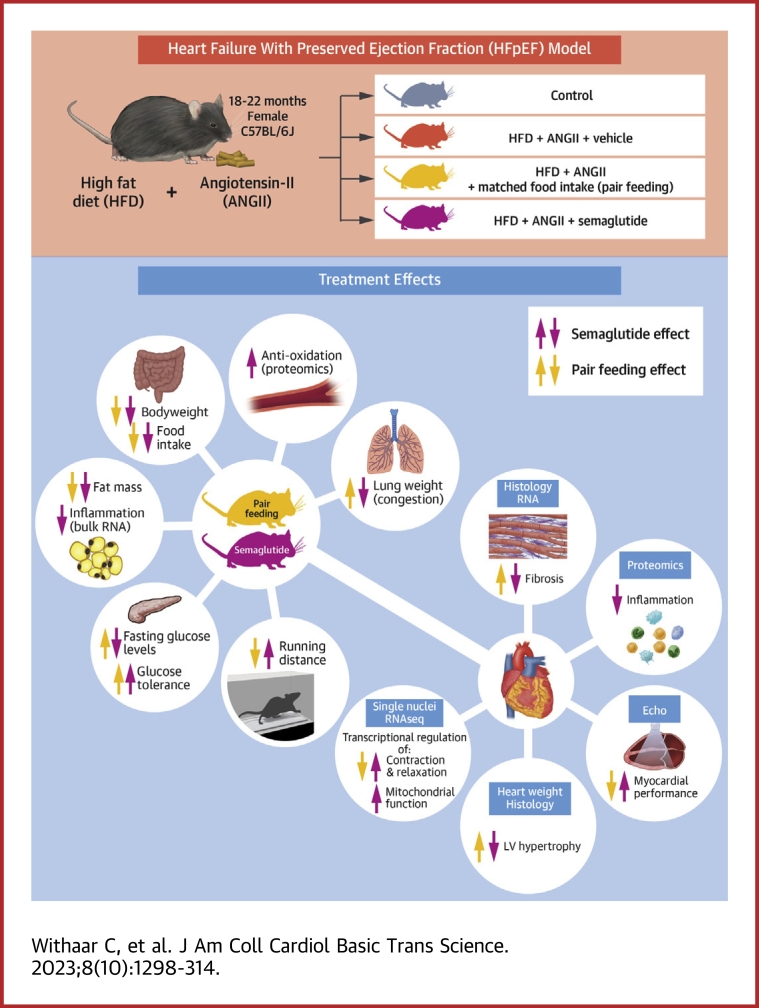

Heart failure (HF) is a major global public health problem. Almost 50% of patients with HF suffer from heart failure with a preserved ejection fraction (HFpEF).^1^ Patients with HFpEF are more often female, elderly, and have multiple comorbidities such as hypertension, type 2 diabetes mellitus (T2DM), and obesity.^2^ To date, ∼80% of all patients with HFpEF have obesity and 20%-45% have T2DM.^3^^,^^4^ As a result of the aging population and increasing prevalence of these comorbidities, HFpEF is expected to become the most common subform of HF.^5^

For a long time, no treatment options to improve outcomes in HFpEF were available, despite a large number of randomized clinical trials being executed.^6^, ^7^, ^8^, ^9^ EMPEROR-Preserved (Empagliflozin Outcome Trial in Patients With Chronic Heart Failure With Preserved Ejection Fraction), evaluating the effects of the sodium glucose co-transporter 2 inhibitor empagliflozin, was the first study to show a significant reduction on a combined “hard” end point of HF hospitalizations and cardiovascular mortality in patients with HFpEF.^10^ Sodium glucose co-transporter 2 inhibitors have metabolic effects, including lowering of plasma glucose levels, and (modest) effects on body weight and blood pressure.^11^ So, these findings validated the concept that effects on the cardiometabolic profile may be effective in HFpEF.^12^^,^^13^

This is in line with data on other interventions that affect body weight. Indeed, marked weight loss achieved through bariatric surgery is associated with improvements in glucose levels and cardiac structure and function in patients with obesity and T2DM.^14^, ^15^, ^16^ Clearly, bariatric surgery results in very substantial weight loss, but ancillary effects also have been described, such as incretin response and changes of the gut microbiome, and some refer to bariatric surgery as metabolic surgery.^17^ Regardless, targeting the unfavorable cardiometabolic profile through weight loss in HFpEF may resemble a powerful treatment strategy to improve outcomes in HFpEF.

A group of drugs that is of particular interest in this respect are the glucagon-like peptide-1 receptor agonists (GLP-1RAs). Long-acting GLP-1RAs exert substantial and sustained body weight losses,^18^ and 2 GLP-1RAs are registered for the indication weight management.^19^ In addition, several GLP-1RAs are approved for effective glucose lowering in patients with T2DM and improve cardiovascular outcomes.^20^^,^^21^ The clinical trials—STEP-HFpEF (Research Study to Investigate How Well Semaglutide Works in People Living With Heart Failure and Obesity; NCT04788511), STEP-HFpEF-DM (Research Study to Look at How Well Semaglutide Works in People Living With Heart Failure, Obesity, and Type 2 Diabetes; NCT04916470), and a dual acting GLP-1/GIP in Participants With Heart Failure With Preserved Ejection Fraction and Obesity; NCT04847557)—are currently recruiting participants with obesity-related HFpEF for treatment with semaglutide or tirzepatide.

Possible mechanisms of action on the cardiovascular system of GLP-1RAs include anti-inflammatory, antiatherosclerotic, vasodilatory, and other hemodynamic effects, and life-long exposure of GLP-1 is associated with prevention of HF.^22^, ^23^, ^24^ Semaglutide treatment results in significant reductions in levels of the inflammatory marker C-reactive protein.^25^ However, it remains elusive what proportion of these effects result from improved glycemic control and significant weight loss and to what extent additional metabolic and cardiovascular effects contribute, transcending reduction in weight.^26^

Therefore, we investigated the cardiometabolic effects of semaglutide in a representative mouse model of HFpEF^27^^,^^28^ and zoomed in on potential underlying actions and molecular mechanisms as compared to pair feeding (PF)-induced weight loss.

## Methods

A detailed methods section is provided in the Supplemental Appendix.

### Data availability

All supporting data are available within this paper and the Supplemental Appendix. Single nucleus RNA-sequencing data have been deposited in the National Center for Biotechnology Information Gene Expression Omnibus and are accessible through GEO series on request. Mass spectrometry data have been deposited to the ProteomeXchange Consortium via the PRIDE (Proteomics Identification Database) partner repository with data set identifier PXD034625.

### Animals

All animal studies were approved by the Centrale Commissie Dierproeven (CCD) license number AVD105002016487 and the Animal Care and User Committee of the Groningen University (permit number 16487-07-04) and conducted in accordance with the ARRIVE (Animals in Research: Reporting In Vivo Experiments) guidelines.^29^ Female C57BL6/J mice, 18-22 months old, were purchased from Jackson Laboratory. Mice were housed on a 12-hour light/12-hour dark cycle with ad libitum access to chow and water. Echocardiography, mini pump placement, and sacrifice were performed using continuous oxygen and 2%-3% isoflurane anesthesia (Teva Pharmaceuticals).^28^

### Experimental design

We have shown previously that a murine model of HFpEF has several similarities with human HFpEF.^27^^,^^28^ Briefly, aged female mice were fed a high fat diet (HFD) (60% kcal fat, 20% kcal protein, 20% kcal carbohydrates; Research Diets D17041409) or a low-fat equivalent control (Ctrl) chow (n = 10; 20% kcal fat, 20% kcal protein, 60% kcal carbohydrates; Research Diets D17041407) for 12 consecutive weeks. After 8 weeks of HFD, mice underwent surgery and an ALZET osmotic mini pump (Model 2004) with angiotensin-II (ANGII) (1.25 mg/kg/d) was implanted in a subcutaneous pocket on the back. For Ctrl mice, the subcutaneous pocket was closed without placement of a pump.

After pump implantation, mice on HFD were assigned to a group with subcutaneous semaglutide treatment (n = 12) or vehicle treatment, phosphate-buffered saline (n = 16) during last 4 weeks of this study. Semaglutide was uptitrated over 5 days to a final dose of 9 nmol/kg/d, and this final dose was continued during 23 days.

**Figure 1:**
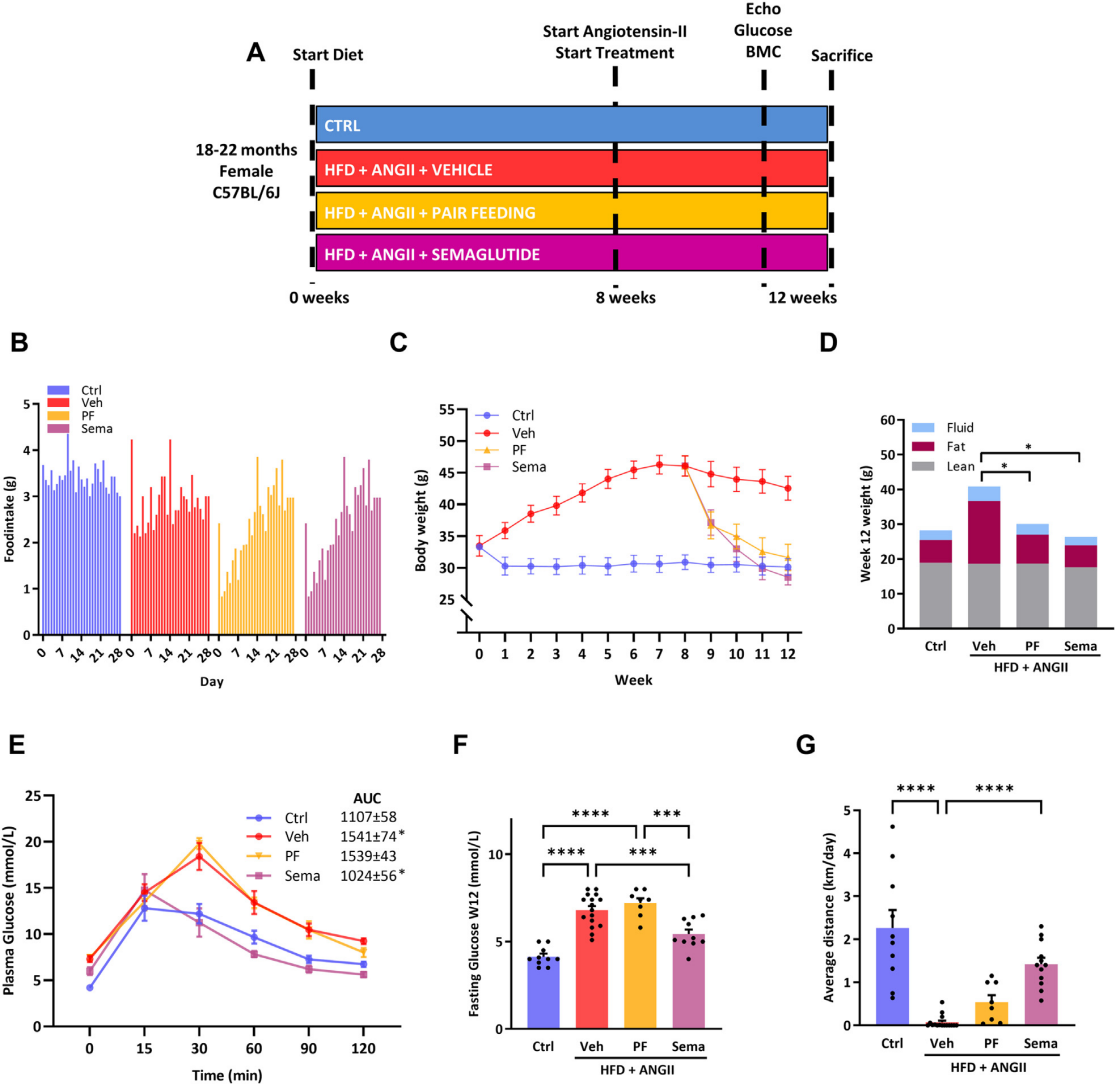
Effects of Sema Treatment or PF on Metabolic Parameters (A) After 12 weeks of high fat diet (HFD) and 4 weeks of angiotensin-II (ANGII) infusion a HFpEF-like fenotpe was induced in aged female mice. The control (Ctrl) group (n = 10) were fed a low fat diet for 12 weeks. After 8 weeks of HFD, mice were assigned to a group with daily injections of semaglutide (Sema) treatment (n=12) or without treatment (daily injections with vehicle [Veh]; n = 16) or pair feeding ([PF]; n = 8) for the last 4 weeks simultaneously continuing with HFD and ANGII. Cardiac measurements (echocardiography) were performed 1 day before sacrifice. Glucose tolerance test, fasting glucose levels, and body mass composition (BMC) were determined prior to sacrifice. (B) Daily food intake during treatment period with Sema, Veh or PF. (C) Weekly body weight for Sema, Veh, or PF. (D) BMC (fluid, fat, and lean mass) measured by minispec LF90II body composition analyzer at week 12. (E) Plasma glucose levels over time (minutes) after glucose loading (2 g/kg body weight) in an oral glucose tolerance test in week 12. (F) Fasting glucose levels per group measured in week 12. (G) Exercise capacity measured by voluntary wheel running. Statistical testing was performed with Kruskal–Wallis test followed by Dunn test. All values are presented as mean ± SEM. *P* < 0.05 was considered statistically significant (∗*P* < 0.05, ∗∗*P* < 0.01, ∗∗∗*P* < 0.001, ∗∗∗∗*P* < 0.0001). AUC = area under the curve.

To determine the extent to which the cardiometabolic effects of semaglutide occurred by changes in body weight or body composition caused by reduced food intake, we designated a separate group of mice, after 8 weeks and pump implantation, to a pair feeding (PF) (n = 8) protocol. With this technique, the amount of food consumed by the semaglutide groups (grams of chow per day) is exactly matched to that consumed by the pair-fed group on a daily basis. A schematic overview of the experimental design is displayed in Figure 1A.

### Exercise capacity

Exercise capacity was determined using voluntary wheel running. After 2 days of acclimatization to the running wheel, we measured running distance during 5 constitutive days in the week prior to sacrifice and the average running distance (km/d) was calculated afterward.

### Fasting glucose and glucose tolerance

After 12 weeks of intervention, mice were subjected to an oral glucose tolerance test. Fasting glucose was measured after 12 hours of fasting followed by administration of an oral bolus of glucose (2 g/kg) and tail vein blood samples were measured using an Accu-Chek Aviva glucose analyzer (Roche Diagnostics) at multiple time points (0, 15, 30, 60, 90, and 120 minutes). Area under the curve was calculated to determine the rate of glucose clearance, as described previously.^28^

### Measurement of body mass composition

Body mass composition (fat mass, fluid, and lean mass) was determined using minispec LF90II body composition analyzer (Bruker Optics) in week 12 according to manufacturer’s protocol.

### Echocardiography

Three days prior to sacrifice, echocardiographic measurements were performed using a Vevo 3100 system equipped with a 40-MHz MXX550D linear array transducer (FUJIFILM VisualSonics) to assess cardiac dimensional and functional parameters. Offline speckle tracking (VEVO strain, Vevo LAB software version 5.5.1) was used to determine global longitudinal strain and reverse peak longitudinal strain rate.^30^

### Tissue procurement and organ morphometry

At week 12, mice were anesthetized with isoflurane and sacrificed. Blood was drawn using apical puncture, collected in EDTA tubes, and spun down, and then the plasma was collected and stored. The heart with all its compartments was rapidly excised, rinsed in ice cold 1 mol/L potassium chloride (Merck Millipore), weighed, and stored for further analyses. Lungs were excised and their wet weight was measured. Visceral adipose tissue (VAT) was collected and stored.

### Histological analysis

Left ventricular (LV) mid-transverse sections were fixed in 4% paraformaldehyde before paraffin embedding and cut into 4-μm thick sections. Masson trichrome stain was performed to detect collagen deposition as a measurement of fibrosis, as previously described.^31^ Cardiomyocyte cross-sectional area (μm^2^) was visualized with immunofluorescent staining with germ agglutinin fluorescein isothiocyanate (Sigma-Aldrich) as previously reported.^28^

### Quantitative real-time polymerase chain reaction

Total RNA from LV tissue was extracted using TRI Reagent (Sigma-Aldrich), and complementary DNA synthesis was performed using the QuantiTect RT kit (Qiagen). Gene expression levels were determined using iQ SYBR green super mix and the CFX384 Touch Real-Time PCR system (Bio-Rad). Expression levels were normalized to reference gene *Rplp0* (36B4), and presented as fold change to the Ctrl group. Primer sequences that were used are displayed in Supplemental Table 1.

### Passive force measurements

Individual cardiomyocytes were isolated mechanically from frozen cardiac tissue, and myofilament function measurements were executed as previously described.^32^ Passive stiffness (Fpassive) was evaluated by increasing sarcomere length of cardiomyocytes from 1.8 μm to 2.4 μm in 0.2-μm steps.

### Plasma ketones measurements

Total ketone bodies were measured in the plasma with the Autokit Total Ketone bodies (FUJIFILM) according to manufacturer’s instructions.^33^

### RNA sequencing

RNA was extracted from VAT tissue using Lysing matrix D beads (MP Biomedicals) and standard TRIzol extraction (Thermo Fisher Scientific). RNA quality was determined using RNA Pico Chips on Bioanalyzer 2100 (Agilent) and TruSeq Stranded mRNA Libraries (Illumina) were generated from high-quality total RNA (RNA integrity number >7.5). Samples were subjected to paired end sequencing with NovaSeq 6000 (Illumina HiSeq). Reads were aligned to mouse reference genome (GRCm38– mm10) using STAR (version 2.7.3a) and read quantification was performed with Salmon (version 1.2.0)^34^ using Ensembl gene annotation GRCm38.101. Principle component analyses and differential expression analysis were performed using DESeq2 (version 1.26.0) Bioconductor. Functional analysis was performed with Gprofiler (e106_eg53_p16_65fcd97, database updated on May 18, 2022; R Foundation). RNA-sequencing data have been deposited in National Center for Biotechnology Information Gene Expression Omnibus and are accessible through GEO series on request.

### Single nucleus RNA sequencing

Single-nucleus suspensions for LV tissue were generated by a series of cellular membrane lysis, differential centrifugation, and filtration steps. Isolated nuclei were loaded into the Chromium Controller (model GCG-SR-1, 10x Genomics) for an estimated recovery of 5,000 cells per sample. Processing of libraries was performed according to the manufacturer’s instructions with few modifications. Samples were sequenced on the NextSeq500/550 Illumina Platform using NextSeq 500/550 High Output Kit (version 2.5, 75 cycles). After sequencing, preprocessing into unique molecular identifier matrices was performed using 10x Genomics Cell Ranger. Ambient messenger RNA was removed with SoupX (Sanger Institute), and cells were assigned a doublet score with Scrublet (Supplemental Figures 5 to 7).^35^ Cell clustering and uniform manifold approximation and projection construction was performed in Seurat, whereas cell type populations were identified using known marker genes (Supplemental Figure 4) and supplemented with automatic cell type annotation SCINA^36^ and scClassify Bioconductor) (Supplemental Figure 5). Differential expression analyses between conditions for every cell population were calculated in Seurat^37^ using the Wilcoxon rank sum test. The R package Gprofiler (g:Profiler version e106_eg53_p16_65fcd97) was used for gene ontology analyses. Full methodologic details for reagents used, nuclear isolation, library construction, quality control, and single nucleus RNA analysis can be found in the extended methods in the Supplemental Appendix.

### Liquid chromatography–tandem mass spectrometry

Total proteins were isolated from LV and plasma followed by denaturation, reduction, alkylation, and tryptic digestion. Peptides were fractionated off-line with an Dionex Ultimate 3000 HPLC system (Thermo Fisher Scientific), and subsequently separated with Easy-nLC 1200 coupled online to an Orbitrap QExactive HF mass spectrometer (Thermo Fisher Scientific). Mass spectrometry data were acquired using a data-independent method as detailed in the extended methods in the Supplemental Appendix. Raw mass spectrometry data were analyzed using the Pulsar search engine (Spectronaut Biognosys). The data were further processed using Perseus software (Maxquant). Enriched protein sets were identified using Gprofiler with significant Gprofiler threshold (*P* < 0.05). Mass spectrometry data have been deposited to the ProteomeXchange Consortium via the PRIDE partner repository with data set identifier PXD034625.

### Statistical analysis

All values are presented as mean ± SEM. Normality of data was tested with Shapiro-Wilk test. Kruskal-Wallis test was used for comparisons among >2 groups followed by Dunn multiple comparisons test with an adjusted *P* value using GraphPad Prism (version 8.42, GraphPad Software) or SPSS (version 23, IBM Corp). The area under the curve was computed using the trapezoid rule. For proteomics, the false discovery rate was set to 0.01, and data are presented as difference of average (>0.5) and the −log_10_ Student’s *t-*test (*P* < 0.05) were used to compare between groups.

*P* < 0.05 was considered statistically significant (∗*P* < 0.05, ∗∗*P* < 0.01, ∗∗∗*P* < 0.001, ∗∗∗∗*P* < 0.0001). For detailed transcriptomic and proteomic statistics, we refer to the extended methods in the Supplemental Appendix.

## Results

### Semaglutide and PF both resulted in weight loss caused by a reduction in fat mass

Treatment with semaglutide was associated with reduced food intake during the first 14 days and normalized afterwards (Figure 1B). Semaglutide resulted in sustained weight loss from first day of treatment onward (Figure 1C). PF resulted in similar weight loss (Figure 1C). In both groups, weight loss was caused by a significant reduction in fat mass in 4 weeks of treatment (19.4-6.2 g for semaglutide; 19.8-8.3 g for PF), as compared to vehicle-treated mice (Figure 1D).

### Semaglutide, but not PF, improved glycemic control and exercise capacity

Treatment with semaglutide, but not PF, improved glycemic control with a significant reduction in fasting glucose levels and improved glucose tolerance, as demonstrated by significantly decreased area under the curve for semaglutide *versus* vehicle-treated mice (Figures 1E and 1F). HFD+ANGII resulted in elevated plasma total ketone bodies, in both this remained with semaglutide treatment and PF (Supplemental Figure 1). Running distance, measured by voluntary wheel running, improved in mice treated with semaglutide and not in mice on PF (Figure 1G).

### Semaglutide improves cardiac function and attenuated LV hypertrophy and lung congestion

**Figure 2:**
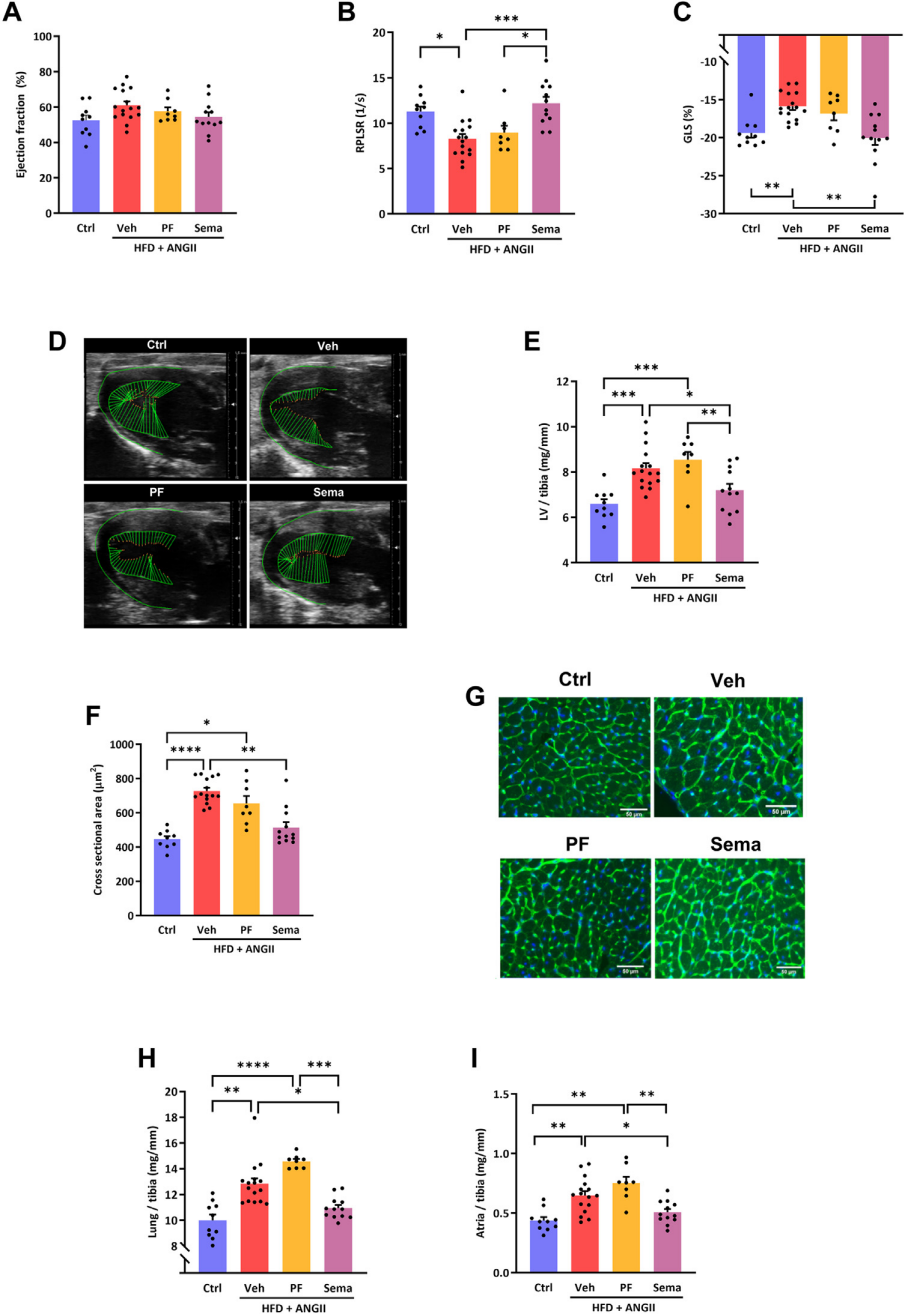
Effects of Sema Treatment or PF on Cardiac Function and Structure Left ventricular ejection fraction (LVEF), measured by echocardiography. (A) Ejection fraction. (B) Global longitudinal strain (GLS) as marker of myocardial deformation. (C) Reverse peak longitudinal strain rate (RPLSR) as parameter of myocardial deformation during early diastole. (D) Representative strain images. (E) Left ventricle (LV) weight corrected for tibia length (LV/tibia). (F) Quantification of cardiomyocyte size. (G) Representative images of cardiomyocyte cell size. (H) Lung weight corrected for tibia length. (I) Atria weight corrected for tibia length. Statistical testing was performed with Kruskal-Wallis test followed by Dunn test. All values are presented as mean ± SEM. *P* < 0.05 was considered statistically significant (∗*P* < 0.05, ∗∗*P* < 0.01, ∗∗∗*P* < 0.001, and ∗∗∗∗*P* < 0.0001). Abbreviations as in Figure 1.

LVEF was preserved in all treatment groups, with no significant differences among groups (Figure 2A). In semaglutide-treated mice, we observed a significant improvement of global longitudinal strain and reverse peak longitudinal strain rate, but this was not the case in mice on PF (Figures 2B to 2D). As compared to PF, semaglutide significantly reduced LV hypertrophy as reflected by reduced LV weight (Figure 2E) and smaller cardiomyocyte size (Figures 2F and 2G), and reduced LV wall thickness on echocardiography (Supplemental Table 2). Furthermore, treatment with semaglutide also reduced atrial and lung weights, which is suggestive of reduced lung congestion (Figures 2H and 2I).

### Treatment with semaglutide reduced cardiac stretch, inflammation, and fibrosis, and this was not related to altered cardiomyocyte stiffness

**Figure 3:**
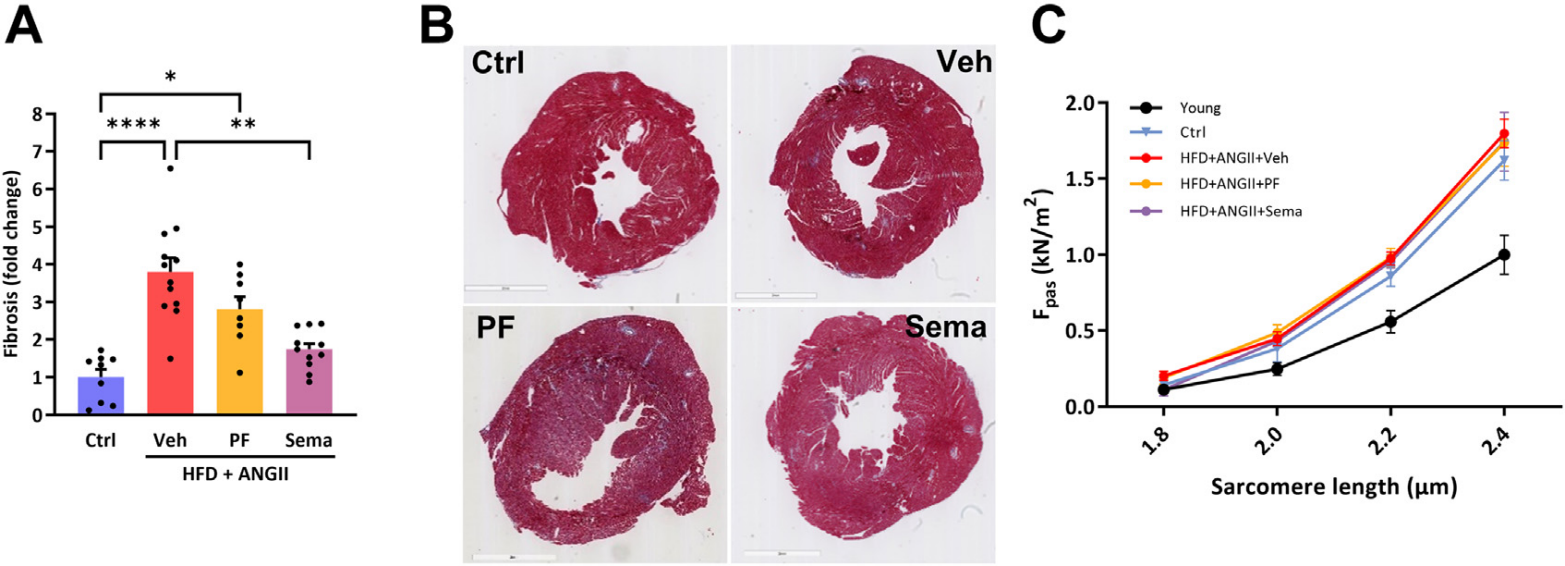
Effects of Sema Treatment or PF on Fibrosis and Cardiomyocyte Stiffness (A) Quantification of Masson staining. (B) Representative images of Masson staining. (C) Intrinsic cardiomyocyte stiffness measured as Fpassive (F_pass_). Statistical testing was performed with Kruskal-Wallis test followed by Dunn test. All values are presented as mean ± SEM. P < 0.05 was considered statistically significant (∗*P* < 0.05, ∗∗*P* < 0.01, ∗∗∗*P* < 0.001, or ∗∗∗∗*P* < 0.0001). Abbreviations as in Figure 1.

In line with the functional improvements, semaglutide treatment significantly reduced messenger RNA levels of cardiac stretch marker (atrial natriuretic peptide) (Supplemental Table 3). It also significantly reduced fibrosis on histologic level (Figures 3A and 3B) and molecular level with significantly reduced messenger RNA levels of collagen 1a1, collagen 3a1, and tissue inhibitor metallopeptidase inhibitor-1 (Supplemental Table 3). Furthermore, semaglutide treatment significantly reduced inflammatory markers such as interleukin-6, galectin-3, and growth derived factor-15. To further assess the functional implications of the reduction in stretch and fibrosis, we then assessed sarcomere stiffness (Fpassive). Increased Fpassive has been observed in HFpEF patient biopsies, and increased cardiac stiffening is associated with aging.^38^^,^^39^ We therefore also added cardiomyocytes of a group of young Ctrl female mice (4 months old). As expected, Fpassive significantly increased with aging. Nevertheless, intrinsic cardiomyocyte stiffness was not affected by any of the interventions, suggesting that in this preclinical model intrinsic stiffness is not involved in HFpEF pathophysiology (Figure 3C).

### In the LV, treatment with semaglutide is associated with improved cytoskeleton function and restored endothelial function

**Figure 4:**
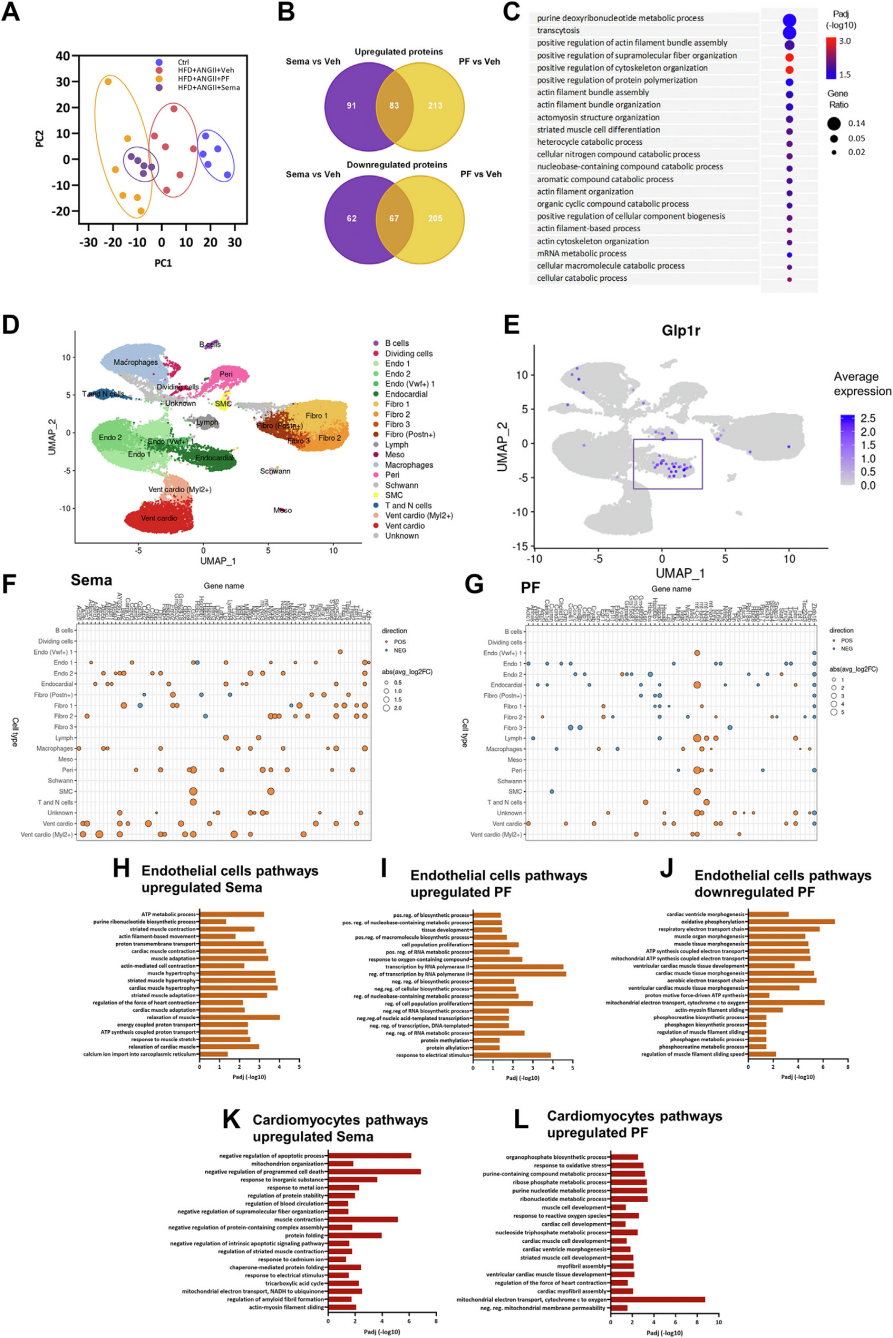
Effects of Sema Treatment or PF on Differently Expressed Proteins, Cell-type–Specific Gene Expression and Pathways in the LV (A) Principal component (PC) analysis of LV identified proteins that uniquely cluster for Ctrl (n = 6), Veh treatment (n = 6), Sema treatment (n = 6), or PF (n = 6). (B) Venn diagram of differently expressed proteins for Sema treatment or PF and their direction vs Veh treatment. (C) Curated Gene Ontology (GO) Biological Processes list of protein-coded genes identified in the LV demonstrating distinctive pathways engaged by Sema treatment vs Veh treatment. Circle size reflects gene ratio, which is the proportion of differentially expressed genes in a pathway divided by all differentially expressed genes that map to GO term. Color-coding reflects *P* value after Gprofiler Threshold (*P* < 0.05). (D) Uniform manifold approximation and projection (UMAP) plot of processed single nucleus RNA-sequencing data from LV tissue of combined Ctrl, Veh, Sema, and PF mice. Coloring indicates manual annotation of cell-type clusters generated by unsupervised clustering. (E) Overlay of glucagon-like peptide -1 receptor (GLP1r) expression onto UMAP. (F, G) Dot plot showing top 10 most-regulated genes per cell type comparing (F) Sema treatment against Veh treatment and (G) PF against Veh. Dot size represents the absolute log_2_-fold change of a gene, whereas the color indicates positive (POS) (orange) or negative (NEG) (blue) regulation of a gene. (H, I) GO enrichment analysis of up-regulated genes in endothelial (Endo) cells comparing (H) Sema against Veh and (I) PF against Veh treatment. (J) GO enrichment analysis of down-regulated genes in Endo cells comparing PF against Veh treatment. (K, L) GO enrichment analysis of up-regulated genes in cardiomyocytes comparing (K) Sema against Veh and (L) PF against Veh treatment. ATP = adenosine triphosphate; Fibro = fibroblasts; Lymph = lymphatic cells; Meso = mesothelial cells; NADH = nicotinamide adenine dinucleotide; Peri = pericytes; SMC = smooth muscle cells; other abbreviations as in Figures 1 and 2.

To better understand the underlying mechanism of semaglutide treatment, we performed proteome analysis of the LV. For this analysis, we compared the effect of semaglutide vs vehicle and the effect of PF vs vehicle. Principal component analysis showed distinct clustering that was more prominent in semaglutide than in mice on PF (Figure 4A). Both semaglutide and PF resulted in a considerable number of uniquely up- or down-regulated proteins (Figure 4B). Using gene set enrichment analyses on up-regulated proteins in semaglutide-treated mice, we identified pathways related to cardiac cytoskeleton processes and actin filament organization (Figure 4C). Of note, these proteomic pathways were not enriched in PF mice (Supplemental Figure 2).

To dissect the semaglutide response in the LV further, we then performed single nucleus RNA sequencing in the LV of mice under conditions similar to the proteomics data. Cell clustering and combined manual and automatic annotation identified 13 major cell types: T cells, natural killer cells, B cells, macrophages, smooth muscle cells, pericytes, endothelial cells, fibroblasts, endocardial cells, Schwann cells, mesothelial cells, lymphatic cells, and ventricular cardiomyocytes, as visualized in a uniform manifold approximation and projection (Figure 4D) (for detailed cell-type annotation per condition see Supplemental Figures 3 and 4). We observed that the main expression of the GLP-1 receptor is in endothelial cells (Figure 4E). Semaglutide or PF resulted in a different cell-specific gene expression pattern (most regulated genes in Figures 4F and 4G and specific regulated genes in endothelial cell types in Supplemental Figure 8). Because of this cell-type–specific expression, and because of the improved structural changes and function of cardiac tissue, we then performed in-depth gene set enrichment analyses on genes regulated in endothelial cells or in cardiomyocytes. We observed that treatment with semaglutide resulted in several enriched pathways in endothelial cells: actin-mediated muscle contraction and relaxation pathways, adenosine triphosphate synthesis and metabolic pathways. This in contrast to PF, where we observed down-regulation of these pathways (Figures 4H to 4J). In cardiomyocytes, semaglutide was associated with multiple pathways enriched for up-regulated genes involved in muscle contraction and mitochondrial function and negative regulation of apoptotic processes. In PF up-regulated genes were enriched, among others, for cardiac muscle cell development (Figures 4K and 4L). Neither semaglutide nor PF down-regulated genes were associated with significant enriched pathways in cardiomyocytes.

### In plasma, treatment with semaglutide differentially regulates pathways related to oxidative-stress processes

**Figure 5:**
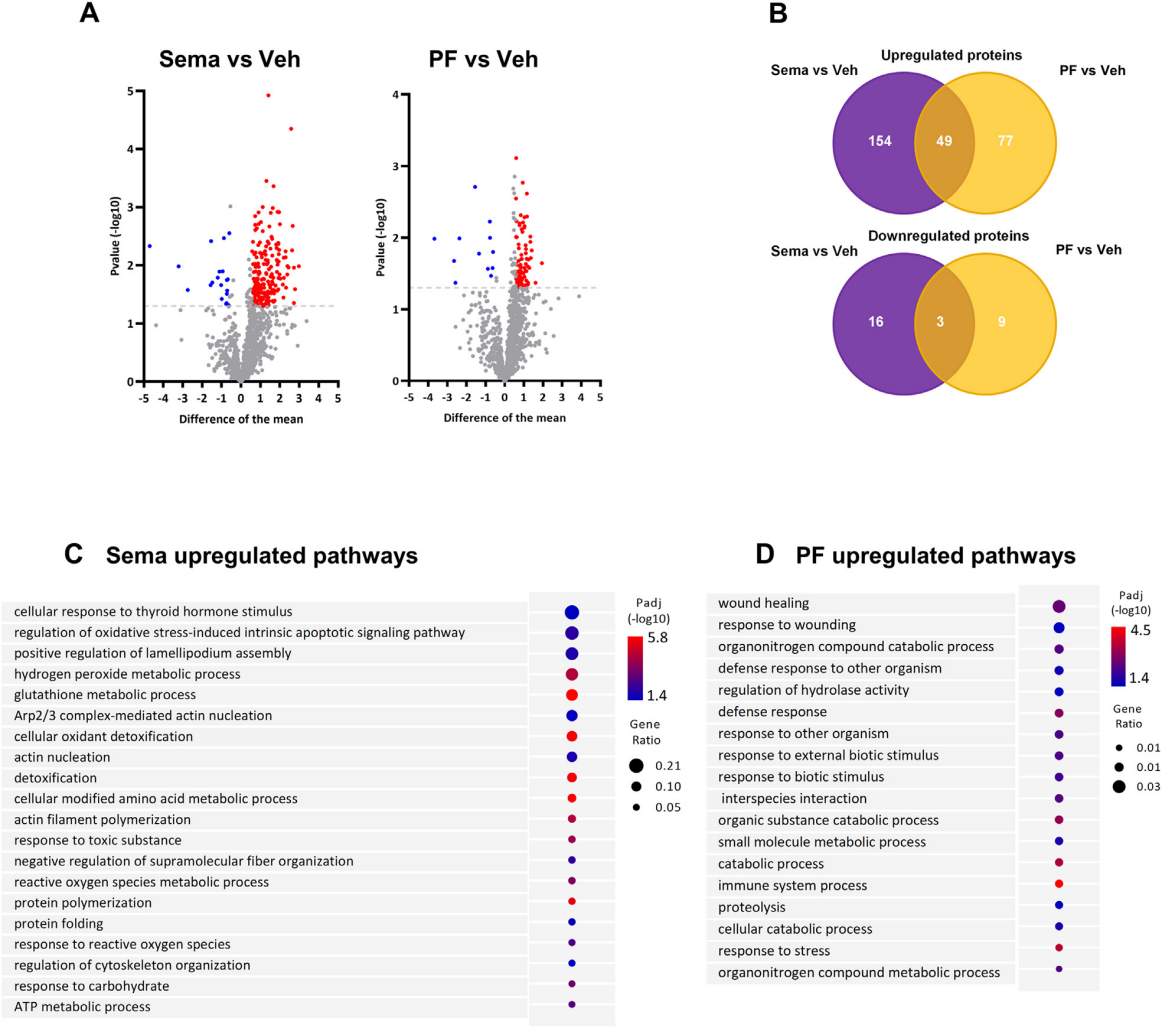
Effects of Sema Treatment or PF on Differentially Expressed Plasma Proteins and Pathways (A) Differentially expressed plasma proteins for Sema treatment or PF compared to Veh treatment. Plasma proteins are presented as average difference >0.5 and the −log_10_ Student’s *t*-test (*P* < 0.05) was used to compare between groups. (B) Venn diagram of differently expressed plasma proteins for Sema and PF and their direction vs Veh treatment. (C, D) Curated GO Biological Processes list of protein identified in the plasma demonstrating distinctive pathways engaged by (C) Sema compared to Veh and (D) PF compared to Veh treatment. Circle size reflects gene ratio, which is proportion of differentially expressed genes in a pathway divided by all differentially expressed genes that map to the GO term. Color-coding reflects *P* value after Gprofiler Threshold (P < 0.05). Abbreviations as in Figures 1 and 4.

We then explored the systemic effects of semaglutide or PF and performed plasma proteomics. A total of 240 proteins were identified with semaglutide treatment and 116 proteins with PF (Figure 5A). Treatment with semaglutide or PF resulted in a clustering of proteins that were significantly altered related to either intervention (Figure 5B). Subsequent functional enrichment analysis in plasma of semaglutide mice identified uniquely up-regulated pathways involved in antioxidant activity and oxidative-stress regulation with related genes—*GPX1, SOD1, PRDX1, PRDX2, GSTP1, GPX3, G6PD*—and cytoskeleton regulation with related genes—*ACTR2, GMFG, ARPC4, ARPC5, RAC1* (Figure 5C). Similar pathways were not observed in mice that were on PF (Figure 5D).

### In VAT, semaglutide activates interferon-γ production, regulates T-cell regulation and T-cell differentiation, and inhibits extracellular matrix organization

**Figure 6:**
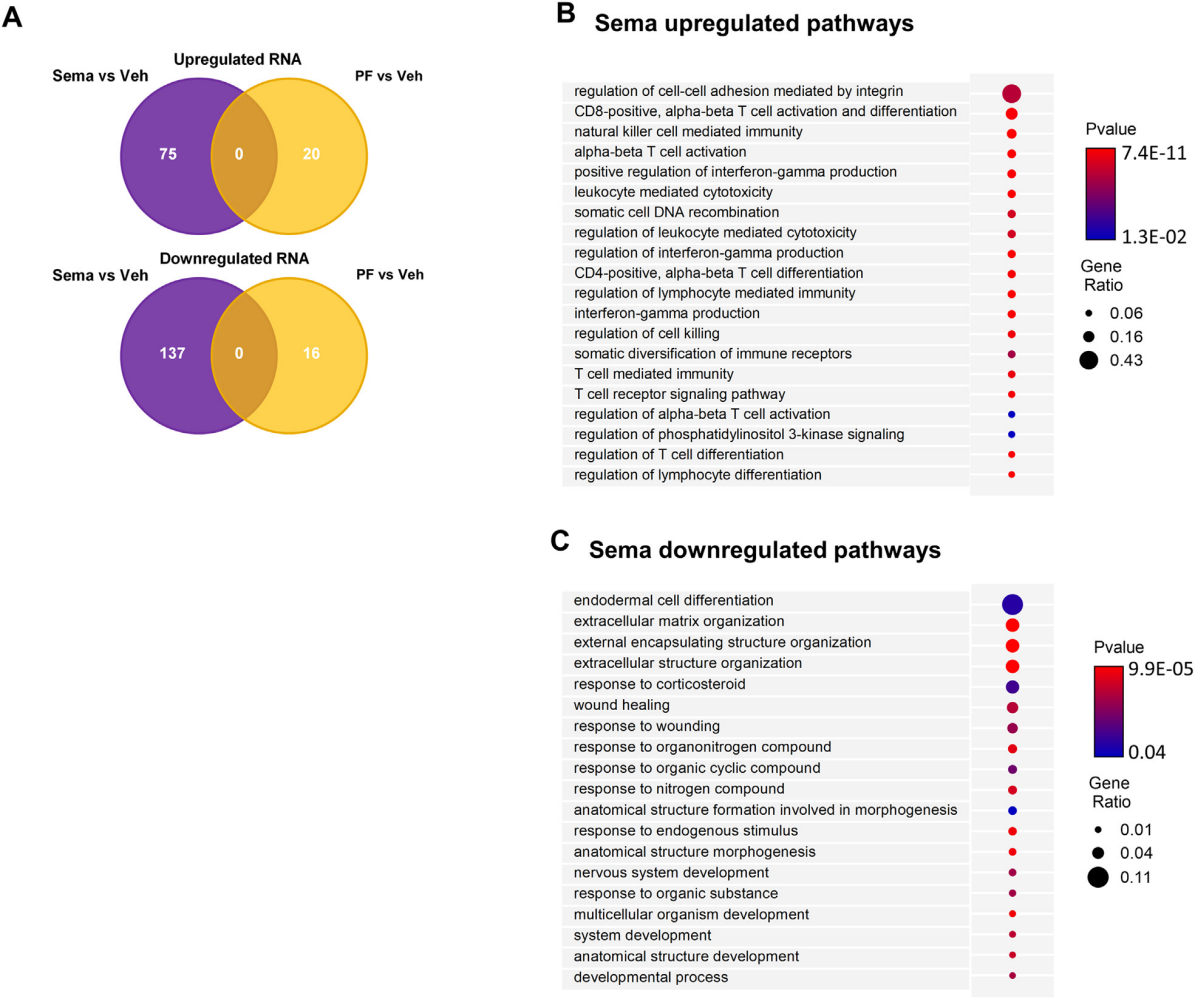
Effects of Sema Treatment or PF on Differentially Expressed VAT Genes and Pathways (A) Venn diagram of differently expressed visceral adipose tissue (VAT) genes for Sema treatment (n = 6) or PF (n = 6) and their direction vs Veh treatment (n = 6). (B, C) Curated GO Biological Processes list of genes identified in the VAT demonstrating unique up-regulated (B) and down-regulated (C) pathways enriched by Sema treatment compared to Veh treatment. Circle size reflects gene ratio, which is proportion of differentially expressed genes in a pathway divided by all differentially expressed genes that map to the GO term. Color-coding reflects *P* value after Gprofiler Threshold (*P* < 0.05). Abbreviations as in Figures 1 and 4.

Because semaglutide and PF both resulted in fat loss, we performed transcriptome analysis on VAT to investigate the effect of weight loss on VAT. We observed that semaglutide had a clear effect on gene expression levels, but this was not the case for mice on PF (Figure 6A). In VAT of mice treated with semaglutide, we identified several uniquely up-regulated pathways involved in T-cell regulation and T-cell differentiation, as well as pathways involved in interferon-γ production (Figure 6B). In addition, in VAT of semaglutide-treated mice, we also uniquely identified down-regulated pathways related to extracellular matrix organization (Figure 6C).

## Discussion

In this study we investigated the effects of the GLP-1RA semaglutide in our established multihit mouse model of HFpEF^28^ and compared the effects to weight reduction in response to PF. We observed that, by design, semaglutide and PF were associated with the exact degree of weight loss mainly caused by fat mass reduction. However, whereas PF only tended to influence some parameters of HFpEF, semaglutide improved several hallmarks of HFpEF, including reduced LV hypertrophy and fibrosis, improved diastolic dysfunction, reduced lung congestion, and improved exercise capacity. Our study demonstrates that the cardioprotective effects of GLP-1RAs in a model of obesity and HFpEF supersede the effects of weight loss by PF alone and provide mechanistic support for the ongoing STEP-HF study of semaglutide in obesity-related HFpEF.

GLP-1RAs, such as semaglutide, are GLP-1 analogs that improve glucose-dependent insulin release by pancreatic βcells, reduce glucagon secretion by αcells, and anti-inflammatory effects.^40^ For this reason GLP-1RAs have traditionally been used for treatment of patients with T2DM to improve glycemic control, for the reduction of cardiovascular events, and slower progression of kidney disease.^23^^,^^41^ GLP-1 also has a direct effect on receptors in the central nervous system and thereby induces weight loss by increased satiety, decreasing hunger and affecting the reward system in various ways leading to better control of food intake. GLP-1RAs are known to induce meaningful weight loss in patients with obesity with or without T2DM.^42^^,^^43^ They attracted strong interest from the cardiovascular field ever since it became clear that GLP-1RAs improve major adverse cardiovascular outcomes including cardiovascular death, myocardial infarction, and stroke in patients with T2DM.^44^^,^^45^ In mechanistic studies, GLP-1RAs were shown to improve cardiac function in patients with T2DM.^21^^,^^46^, ^47^, ^48^, ^49^, ^50^, ^51^, ^52^ It had already been demonstrated that treatment with GLP-1RAs activates several cardioprotective pathways, prevents HFD-induced insulin resistance and inflammation, improves endothelial function, and ameliorates cardiac function in obese mice.^53^ In this study, therefore, we conducted detailed studies using single-cell transcriptomics and proteomics of the LV, adipose tissue, and plasma to identify potential underlying mechanisms through which the GLP-1RA semaglutide may improve cardiac function and cardiovascular outcomes in patients with HFpEF. Furthermore, we compared the effects and pathways to the most common intervention to combat obesity-induced HFpEF—dietary measures such as PF.

Several studies reported that (modest) weight loss caused by lifestyle interventions in obesity-related HFpEF patients improved exercise capacity but had no effect on cardiac structure or function.^54^, ^55^, ^56^, ^57^ A modest weight loss may therefore not be enough to improve cardiac structure and function, because a significant reduction of >10% in VAT and epicardial adipose tissue is needed to achieve a sustained effect.^58^^,^^59^ Furthermore, weight loss by lifestyle interventions is notoriously difficult to achieve and rarely sustained for a longer period. Extreme measures such as bariatric surgery come with considerable risks and side effects such as infections, bleeding, and absorption problems. Therefore pharmacologic treatment may be an effective and safe way of achieving long-term sustained weight loss. Results from STEP 1 (Research Study Investigating How Well Semaglutide Works in People Suffering From Overweight or Obesity) demonstrated that once weekly subcutaneous injection with semaglutide resulted in a marked and sustained weight loss of >15% in patients who are overweight or obese.^7^ This is in line with the results of our experimental study, in which we also observed a reduction in fat mass (>15%) but not in lean mass.

In our study, we used a PF protocol to match the semaglutide-induced reduction in food intake, with subsequent weight loss, to find out whether semaglutide exerts additional drug-induced effects beyond weight loss alone. Semaglutide resulted in an ∼60% food reduction in week 1, ∼30% in week 2, and ∼10% in weeks 3 and 4. Matching this food intake, however, was not enough to improve glucose homeostasis in our PF group, as observed with other (true) caloric restriction protocols used in mice. Pak et al^60^ showed that prolonged fasting of 10 months resulted in caloric restriction–mediated increase in insulin sensitivity. Furthermore, long term >40% caloric restriction recovered insulin sensitivity, fasting glucose, and insulin levels,^61^^,^^62^ leading us to believe that our protocol was not long enough to observe these effects in the PF group.

A distinct feature of HFpEF is the presence of multiple comorbidities and together with hypertension and aging, obesity is a main risk factor for HFpEF. Obesity indirectly promotes insulin resistance and hyperglycemia and has additional direct deleterious effect, given that adipose tissue is highly metabolically active and involved in promoting a systemic proinflammatory state.^63^ Accumulating evidence emerged that dysfunction of endothelial cells, induced by hyperglycemia and driven by this systemic proinflammatory state, trigger cardiomyocyte dysfunction in HFpEF.^64^, ^65^, ^66^ Several animal studies and studies with patients with T2DM have already shown that GLP-1RAs have anti-inflammatory and antioxidant properties, and this may reflect a pathway by which semaglutide exerts (a part of) its effects.^26^^,^^67^^,^^68^ In this study we validated these anti-inflammatory and antioxidant effects: in plasma, semaglutide—but not PF—increases antioxidant enzymes that protect against oxidative stress damage; such as glutathione peroxidase, peroxiredoxin, and catalase. In VAT, semaglutide significantly enriched pathways related to protective inflammatory responses, whereas PF—which was able to reduce fat content in a similar manner—did not have these additional positive effects. Therefore, this study clearly demonstrates the favorable cardiometabolic effects of GLP-1RAs are not solely the result of altered glucose metabolism or reduced VAT volume, but they also result from reduced inflammatory and oxidative stress state.^66^^,^^69^

Inhibition of systemic metabolic inflammation with glucose-lowering drugs is thought to enhance endothelial and cardiomyocyte function,^70^, ^71^, ^72^ because continuous myocardial exposure to high glucose levels induces advanced glycation of contractile proteins and causes alterations in cardiac actin and myosin cytoskeleton that are associated with decreased LV function.^73^ In our HFpEF animal model, we show that the GLP-1 receptor is mainly present in endothelial cells and not in cardiomyocytes, suggesting that GLP-1RAs do not directly target cardiomyocytes. Recent work by Helmstadter et al^74^ showed that the endothelium-localized GLP-1 receptor indeed appears to be responsible for the positive effects of GLP-1RAs. In our study we demonstrated that consistent in both -omics data sets, treatment with semaglutide results in enriched pathways related to cytoskeleton organization and mitochondrial function in endothelial cells. We therefore hypothesize that the effects of semaglutide on cardiac function and cardiac structure are primarily the result of improved endothelial function and secondarily to that a possibly improved muscle contraction caused by intimate cell-cell interactions within the heart.^75^

Myocardial diastolic stiffness is among the hallmarks of HFpEF and results from both myocardial fibrosis and increased cardiomyocyte stiffness. Targeting these 2 components of myocardial diastolic stiffness may therefore be an attractive therapeutic intervention for HFpEF. In this study we observed that semaglutide improved cardiac structure and reduced LV hypertrophy through reduced fibrosis and fibrosis mediators. Other vitro studies observed a beneficial effect of GLP-1RAs on ANGII-induced cardiac fibrosis as well.^53^^,^^74^^,^^76^^,^^77^ The effects of GLP-1RAs are not organ-specific and a reduction in fibrosis and improved outcome have also been seen in patients with diabetic kidney disease.^78^ This suggests that GLP-1RAs may have specific antifibrogenic properties in several organs, including the heart.

Cardiomyocyte stiffness refers to changes in relaxation and filling properties and includes alterations in myofilament deactivation by titin (a giant sarcomeric protein)^79^ or in acto-myosin kinetics.^80^ In our study, treatment with semaglutide resulted in improved cardiac function with less myocardial diastolic stiffness. We observed that semaglutide had no effect on sarcomere stiffness (Fpassive) but resulted in enrichment of actin-myosin and muscle contraction pathways. This suggests that the functional improvement is rather a result of improved actomyosin kinetics than altered sarcomere stiffness.

### Study limitations

In this study we used a multifactorial preclinical HFpEF model that resembles the cardiometabolic human HFpEF phenotype to a large extent. Although this model includes aging, obesity, impaired glucose handling, and female sex, the model is still not representative of the entire spectrum of HFpEF. The number of comorbidities in humans with HFpEF often is even larger, and direct translation of our findings into clinical practice must be done cautiously. Also, we only used aged female mice with HFpEF. In humans, the HFpEF phenotype is more prevalent in aging women and less common in aging men,^81^ but future studies are needed to investigate whether semaglutide has potential sex-specific effects. Semaglutide was administered in a phase where the HFpEF in part developed, namely after 8 weeks of HFD and at the start of ANGII infusion. Therefore, our timing does not allow us to fully dissect whether semaglutide would prevent incident HFpEF or rather would attenuate prevalent HFpEF or both. In addition, unraveling the role of GLP-1 receptor located on endothelial cells in the context of aging and comorbidities are needed but require lengthy experiments with old animals that require follow-up studies.

### Clinical implications and translational outlook

Given their promising cardiovascular effects in patients with T2DM, GLP-1RAs, including semaglutide, are currently being evaluated in patients with HFpEF. However, it remains unclear whether the potential effects of semaglutide are pathway- and drug-specific or whether they are indirectly caused by weight loss. Here, we evaluated the cardiometabolic effects of semaglutide in a validated HFpEF mouse model. We show that the cardiometabolic effects of semaglutide transcends those of weight loss alone, and we demonstrate that semaglutide induces structural and functional changes in the heart and adipose tissue that are associated with improved cardiac structure and cardiac function. Therefore, this study provides mechanistic evidence that semaglutide may represent an effective novel therapeutic option for patients with obesity-related HFpEF.

## Conclusions

HFpEF is a major and growing public health problem.^82^ To date, treatment options for HFpEF are limited and novel treatment options are eagerly awaited. Targeting the unfavorable cardiometabolic phenotype may represent an attractive treatment strategy, especially for those patients with obesity-related HFpEF. Our study shows that the GLP-1RA semaglutide has numerous cardiometabolic effects that extend beyond the effects of weight loss per se. Clinical studies—STEP-HFpEF (NCT04788511) and STEP-HFpEF-DM (NCT04916470)^83^—are already ongoing to address whether semaglutide will improve function in HFpEF, and SELECT (Semaglutide Effects on Heart Disease and Stroke in Patients With Overweight or Obesity; NCT03574597) addresses whether semaglutide affects cardiovascular outcomes in patients with obesity. Preclinical studies such as this study help in understanding the mechanistic basis for observed clinical results.

In conclusion, we have shown that in a multifactorial experimental animal model of HFpEF, the cardiometabolic effects of semaglutide extend beyond the effects of weight reduction caused by PF alone. Our results provide mechanistic evidence that GLP-1RAs, particularly semaglutide, could be a potential novel therapeutic option for patients with obesity-related HFpEF.

Perspectives **COMPETENCY IN MEDICAL KNOWLEDGE:** This study provides insights into the potential use of GLP-1RAs as body-weight and adipose-tissue modifying treatment options, and how they may have additional beneficial effects on the cardiometabolic profile, cardiac structure and cardiac function in obesity-related HFpEF.

**TRANSLATIONAL OUTLOOK:** The present study is the first study to show that the GLP-RA semaglutide may be potentially useful for the treatment of patients with obesity-related HFpEF.

## Funding Support and Author Disclosures

This study was supported in part by Novo Nordisk, the manufacturer of semaglutide, who financed the laboratory supplies and -omics studies. The University Medical Center Groningen, which employs several of the authors, has received research grants and/or fees from AstraZeneca, Abbott, Boehringer Ingelheim, Cardior Pharmaceuticals GmbH, Ionis Pharmaceuticals, Inc, Novo Nordisk, and Roche. Dr Lam has received support from a Clinician Scientist Award from the National Medical Research Council of Singapore; has received research support from Bayer and Roche Diagnostics; has served as consultant or on the Advisory Board/Steering Committee/ Executive Committee for Abbott, Actelion, Alleviant Medical, Allysta Pharma, Amgen, AnaCardio AB, Applied Therapeutics, AstraZeneca, Bayer, Boehringer Ingelheim, Boston Scientific, Cytokinetics, Darma Inc, EchoNous Inc, Impulse Dynamics, Ionis Pharmaceutical, Janssen Research and Development LLC, Medscape/WebMD Global LLC, Merck, Novartis, Novo Nordisk, Prosciento Inc, Radcliffe Group Ltd, Roche Diagnostics, Sanofi, Siemens Healthcare Diagnostics, and Us2.ai; and is a co-founder and a nonexecutive director of Us2.ai. Dr de Boer has received speaker fees from Abbott, AstraZeneca, Bayer, Novartis, and Roche. All Novo Nordisk authors disclose the company markets semaglutide for the treatment of diabetes and obesity, separately, and have multiple clinical studies ongoing. Also, all Novo Nordisk authors report they have minor amount of shares as part of employee benefits. All other authors have reported that they have no relationships relevant to the contents of this paper to disclose.
