# Supplementary material for: The Cardioprotective Effects of Semaglutide Exceed Those of Dietary Weight Loss in Mice With HFpEF
Source: JACC Basic Transl Sci. 2023 Jul 26;8(10):1298–314. doi: 10.1016/j.jacbts.2023.05.012 (PMC10714176; doi:10.1016/j.jacbts.2023.05.012)
Supplement: Supplemental Material [file mmc1.pdf]

SUPPLEMENTAL MATERIAL

The cardioprotective effects of semaglutide exceed those of dietary weight loss in mice with HFpEF

Authors:

Coenraad Withaar Msc[2*], Laura M.G. Meems PhD MD[2*], Edgar E. Nollet MSc[3,4], E. Marloes Schouten[2], Marie A. Schroeder PhD[6], Lotte B. Knudsen PhD[6], Kristoffer Niss PhD[6], Christian T. Madsen PhD[6], Annabelle Hoegl PhD[6], Gianluca Mazzoni PhD[6], Jolanda van der Velden PhD[3,4], Carolyn S.P. Lam PhD MD[2,5], Herman H.W. Silljé PhD[2], Rudolf A. de Boer, PhD MD[1,2]

[1] Erasmus MC, department of Cardiology, Dr. Molewaterplein 40, 3015GD, Rotterdam, the Netherlands

[2] University of Groningen, University Medical Center Groningen, Department of Cardiology, The Netherlands, Hanzeplein 1, 9713 GZ, Groningen, the Netherlands

[3] Amsterdam UMC location Vrije Universiteit Amsterdam, Physiology, De Boelelaan 1117, Amsterdam, the Netherlands

[4] Amsterdam Cardiovascular Sciences, Heart failure & arrhythmias, Amsterdam, The Netherlands

[5] National Heart Centre, Singapore, and Duke-National University of Singapore

[6] Research and Early Development, Novo Nordisk A/S, Denmark

* equal contribution

Supplementary Data

1. Extended methods

1.1 Animals

All animal studies were approved by the Central Committee of Animal experiments (CCD) license number AVD105002016487 and the Animal Care and User Committee of the Groningen University (permit number 16487-07-04) and conducted in accordance with the ARRIVE guidelines [30], the general principles governing the use of animals in experiments of the European Communities (Directive 2010/63/EU) and Dutch legislation (The revised Experiments on Animals Act, 2014). Female, 18-22 months old C57BL6/J mice were purchased from Jackson laboratory. Mice were housed on a 12h light/12h dark cycle with *ad libitum* access to chow and water. Echocardiography, mini pump placement, and sacrifice were performed under anesthesia with oxygen and 2-3% isoflurane, as published before [29]. Animals were euthanized by dissecting the diaphragm under isoflurane anesthesia, after which organs were harvested.

1.2 Experimental design

We have shown previously that a murine model of HFpEF has several similarities with human HFpEF.[28,29] Briefly, aged female mice were fed a high fat diet (HFD; 60% kcal fat, 20% kcal protein, 20% kcal carbohydrates; Research Diets D17041409), or a low-fat equivalent chow (CTRL, n = 10; 20% kcal fat, 20%kcal protein, 60% kcal carbohydrates; Research diets D17041407) for 12 consecutive weeks. After 8 weeks of HFD, mice underwent surgery and an ALZET® osmotic mini pump (Model 2004) with Angiotensin-II (ANGII) (1,25 mg/kg/day) was implanted in a subcutaneous pocket on the back. For CTRL mice, the subcutaneous pocket was closed without placement of a pump.

After pump implantation, mice on HFD were assigned to a group with subcutaneous (s.c.) semaglutide treatment (Sema, n = 12) or Vehicle treatment, PBS (Veh, n = 16) during last four weeks of this study. Semaglutide was uptitrated over 5 days to a final dose of 9 nmol/kg/day and this final dose was continued during 23 days. To determine the extent to which the cardiometabolic effects of semaglutide occurred by changes in body weight or body composition caused by reduced food intake we designated a separate group of mice, after 8 weeks and pump implantation, to pair feeding (PF, n = 8) using a pair-feeding protocol. With this technique the amount of food consumed by the semaglutide groups is matched to that consumed by the pair-fed group on daily base. A schematic overview of the experimental design is displayed in figure 1A.

1.3 Echocardiography

Three days prior to sacrifice, 2D transthoracic echocardiographic measurements were performed using a Vevo 3100 system equipped with a 40-MHz MXX550D linear array transducer (FUJIFILM VisualSonics). Prior to echocardiographic imaging, mice were anesthetized (2% isoflurane (TEVA Pharmachemie) mixed with oxygen, administered via an aerial dispenser) and the hair was removed from the thorax using a commercially available topical depilation agent with potassium thioglycolate (Veet). Mice were fixed in supine position on the (37°C) temperature-maintained platform of the Vevo imaging station (FUJIFILM VisualSonics) over the integrated electrode pads to monitor the heart and respiration rates. Parasternal short and long axis views were obtained at Left Ventricular (LV) mid-papillary level. Vevo LAB software (version 5.5.1) was used to assess cardiac dimensional and functional parameters and offline speckle tracking analysis (VEVO strain) was used to determine myocardial performance as previously described.[29,31,78] Parasternal long axis Bmode recordings were

used for speckle-tracking analysis to evaluate global longitudinal strain (GLS) and reverse peak longitudinal strain (RPLSR) by outlining the epicardial and endocardial borders. Short axis M-mode recordings were used to determine heart rate, LV end-diastolic internal diameter (LVIDd), LV end-systolic internal diameter (LVIDs), left ventricle anterior wall thickness (LVAW), left ventricle posterior wall thickness (LVPW) and fractional shortening (FS) by outlining the epicardial and endocardial borders using the LV Trace tool. For two-dimensional (2D) measurements, three subsequent cardiac cycles, unaffected by respiration, were analyzed. A blinded observer performed all measurements.

1.4 Histological analysis

LV mid-transverse sections were fixed in 4% paraformaldehyde before paraffin embedding, as described previously[79] and cut into 4µm thick sections. Masson's trichrome stain (Masson) was performed to detect collagen deposition as a measurement of fibrosis as previously described.[32] Cardiomyocyte cross sectional area (CSA) (µm^2) was visualized with immunofluorescent staining with germ agglutinin fluorescein isothiocynatae (WGA-FITC) (Sigma-Aldrich) as previously reported.[29] For cardiomyocyte size quantification, five randomly selected fields from WGA-FIT,C LV sections imaged at 20X magnification were used to measure cross-sectional diameter from approximately 50 cells per mouse and calculated as average cross sectional area (CSA).

1.5 Passive force measurements

Individual cardiomyocytes were isolated mechanically from frozen cardiac tissue and myofilament function measurements were executed as previously described.[33] Briefly, frozen tissues were thawed and mechanically disrupted in relax solution containing 1 mM Mg2+, 145 mM KCl, 2 mM EGTA, 4 mM ATP, 10 mM imidazole (pH 7.0). Membranes were permeabilized with 0.5% Triton-X-100, after which individual cells were mounted to a motor needle and a force transducer needle. Passive stiffness was evaluated by increasing sarcomere length of cardiomyocytes from 1.8 µm to 2.4 µm in 0.2 µm steps. Maximal force generation was measured by transferring cardiomyocytes to activating solution with a pCa of 4.5.

1.6 Visceral adipose tissue (VAT) RNA sequencing

RNA was extracted from pulverized VAT tissue using Lysing matrix D beads (MP Biomedicals) and standard TRIzol extraction (Thermo Fisher Scientific). RNA quality was determined using RNA Pico Chips on Bioanalyzer 2100 (Agilent) and TruSeq stranded mRNA libraries (Illumina) were generated from high quality total RNA (RNA integrity number > 7.5). Samples were subjected to paired end sequencing with NovaSeq 6000 (illumine HiSeq). Reads were aligned to mouse reference genome (GRCm38 - mm10) using STAR 2.7.3a and read quantification was performed using Salmon 1.2.0. using Ensembl gene annotation GRCm38.101. Principle component analyses (PCA) and Differential Expression (DE) analysis were performed using DESeq2 1.26.0. Only protein coding genes with at least 1 CPM were used in the analysis (18637 genes). Gene functional enrichment was performed using Gprofiler 2.0.2.0 (e106_eg53_p16_65fcd97, database updated on 18/05/2022) separately for up-regulated and down-regulated sets of genes. Revigo was used to reduce redundant pathways and only unique pathways for Sema or PF were showed. RNA-Seq data have been deposited in NCBI Gene Expression Omnibus and are accessible through GEO Series upon request.

1.7 Left Ventricle single nuclei RNA sequencing

1.7.1 Single nuclei isolation

Single-nuclei suspensions were obtained from snap frozen cryo-grinded tissue by further homogenization and cellular membrane lysis by the use of lysis buffer (Nuclei Extraction Buffer,

Miltenyi biotec* + 0.2 U/µl RNase Inhibitor Roche) in combination with the gentleMACS™ Octo Dissociator with Heaters (Miltenyi biotec). For single nuclei isolation each group consist of n = 3 pooled samples (pooled 3 animals). Homogenate was then filtered through MACS® SmartStrainers 30 µm (Miltenyi biotec) and pluriStrainer®Mini 10 µm (pluriSelect Life Science) and followed by centrifugation (300 x g, 10 min, 4°C). Supernatant was removed and pellet was resuspended in Resuspension Buffer (DPBS -Ca2+, -Mg2+, Sigma-Aldric + 0.4% BSA, Sigma-Aldrich + 0.2 U/µl RNase Inhibitor, Roche). A debris removal step was added using Debris Removal Solution (Miltenyi biotec) following supplier's protocol. After a final centrifugation step the pellet was resuspended in resuspension buffer and nuclei were counted on NucleoCounter NC-200 (ChemoMetec, average of two counts performed) and visually inspected under microscope. No FACS sorting was performed for the single-nuclei suspensions to be as un-biased as possible in regard to cell populations.

1.7.2 cDNA Library preparation

Isolated nuclei were loaded into the Chromium Controller (Model GCG-SR-1, 10X Genomics) with a Targeted Cell Recovery set to 5000 cells per sample. cDNA Libraries were generated according to supplier's instructions (10X Genomics) with a few modifications using the Chromium Next GEM Single Cell 3'GEM, Library & Gel Kit, v3.1 (10X Genomics) with a Single Index T Set A kit (10X Genomics) and steps performed on the C1000 Touch™ Thermal Cycler with a 96–Deep Well Reaction Module (Bio-Rad)

1.7.3 Single-nuclei RNA sequencing

cDNA libraries were combined two and two (2nM each cDNA Library) and sequenced on the NextSeq500/550 Illumina Platform using NextSeq 500/550 High Output Kit v2.5 (75 Cycles, Illumina). A Phix control (Illumina) was included in a final concentration of 1%.

1.7.4 Sample Processing and Quality Control

Samples representing different groups were processed together in series of either 2 or 4 samples from single-nuclei suspensions to construction of cDNA library. Samples were loaded in random order on the Chip G, across the lanes of the chip, thereby preventing any possible technical bias of the groups. Samples quality checked on Qubit 4 Fluorometer (Invitrogen) using the Qubit TM 1X dsDNA HS Assay Kit (Invitrogen) and on the 2100 Bioanalyzer (Agilent) using the Agilent High Sensitivity DNA Kit. Sequencing quality was verified by the Q30 value after each run (Q30 all in the range 90.8-95.3%).

1.7.5 Genome alignment, gene expression quantification, doublet scoring and ambient mRNA cleaning

Samples were processed into count matrices with Cell Ranger from 10x Genomics (mkfastq v4.0 and count v6.0.1) using default values and setting include introns to True. As reference genome, we used the mouse reference mm10 (GENCODE vM23/Ensembl 98) provided by 10x Genomics for Cell Ranger (https://support.10xgenomics.com/single-cell-gene-expression/software/release-notes/build). Each cell was assigned a doublet score using Scrublet (v0.2.3) (expected doublet rate 0.06, min counts 2, min cells 3, min gene variability pct 85) on the filtered count matrices. The R package SoupX with default parameters was used to adjust for ambient mRNA, being provided the filtered and unfiltered count matrices and the graph-based clustering produced by Cell Ranger.

(using hearth and aorta scRNA-Seq data from Tabula Muris)

1.7.6 UMAP construction and cell clustering

SoupX-cleaned count matrices was further processed using the SCTransform-based workflow of Seurat (v4.1). Cells were filtered by nFeature_RNA > 250, nFeature RNA < 9000, percent.mt < 5, percent.ribo < 5 and scrublet score < 0.2. For the SCTransform function, the variables nCount_RNA, orig.ident (sample origin), percent.mt and percent.ribo were regressed. 20 PCA dimensions were used for both FindClusters and RunUMAP, while a clustering resolution of 0.5 was used for FindClusters. For further down-stream analysis, the RNA assay was used and normalized using NormalizeData.

1.7.7 Cell type identification

Manual cell type annotation of clusters was based on both human and mouse gene markers from.[83–85] The manual annotation was supplemented with automatic cell type annotation using SCINA (using hearth and aorta scRNA-Seq data from Tabula Muris) with default parameters and a reference list of markers. In addition, we used scClassify with default parameters based on the heart and aorta dataset from tabula muris.[86]

1.7.8 Differential expression and gene ontology analysis

For the in-depth analysis of the ventricular and endothelial cells, we used return.thresh 0.05, logfc.threshold 0.25, min.pct 0.25, min.diff.pct 0.10 and finally filtering for adjusted p value < 0.05. Gene functional enrichment was performed using Gprofiler (e106_eg53_p16_65fcd97, database updated on 18/05/2022) separately for up-regulated and down-regulated sets of genes. Revigo was used to reduce redundant pathways and only unique pathways for Sema or PF were showed with focus on biological process gene ontologies, with significant g:SCS Threshold p < 0.05.

1.8 Proteomics

1.8.1 Left ventricle tissue processing

Total protein was isolated from frozen left ventricular tissues by adding 8 µL ice-cold buffer; 2% sodium deoxycholate (SDC), 5 mM tris (2-carboxyethyl)phosphine (TCEP), 10 mM chloroacetamide (CAA), 5 mM EDTA, 10 mM KCl, 100 mM triethylammonium bicarbonate (TEAB), pH 8.5 per mg of tissue. The samples were homogenized using the TissueLyzer II (Qiagen, Hilden, Germany) at 30 Hz using 3 mm zirconium beads (OPS Diagnostics). After homogenization samples were sonicated at 4°C using a cuphorn (Qsonica) using 3x 4 second on-time (75 amplitude) with a pulse-off time of 10 seconds. Samples were incubated at 95°C at 650 rpm, and then centrifuged at 15,000 g for 20 min at 4°C. Protein concentration was determined using a Nano-drop (Thermo Scientific) at 280 nm. Protein (500 µg pr. sample) was digested overnight at 37°C using a Trypsin / Lys-C mix (Promega) at 1:200 (w/w) dilution. Digestion was quenched by adding equal volume of 0.1% formic acid, 1% trifluoroacetic acid (TFA), and centrifuged at 14,000 g for 10 min. The supernatant was carefully transferred to a new tube without disturbing the SDC precipitate, and the digest cleaned on a prewashed C18 SepPak (Waters, USA). Peptides were eluted using 400 µl 40% acetonitrile (ACN) and 400 µl 60% ACN, subsequently dried in a SpeedVac centrifuge and reconstituted in 65 µl 5 mM ammonium bicarbonate.

1.8.2 Plasma sample processing

For library generation, two protein depletion kits (Pierce Top 14 abundant protein immunodepletion kit A3637 and BioRad Proteominer kit 1633006) were used separately and the results were combined to establish a spectral library. For sample preparation, 10 µL of plasma was depleted using Pierce Top 12 abundant protein immunodepletion kits (85165). All kits were used according to the manufacturer protocol. Upon Proteominer use, proteins were precipitated using Trichloracetic acid (TCA) at 20% final concentration and washed with cold acetone to remove detergents and highly concentrated

denaturants from the buffer prior to reduction, alkylation, and digestion. Proteins were reduced and alkylated in Phosphate-buffered saline (PBS) containing 1% SDC, 10 mM TEAB pH 8.5, 5 mM TCEP and 10 mM CAA by heating for 10 min at 90°C. After cooling down to room temperature, the proteases trypsin (Sigma Aldrich) and LysC (Wako) were each added in an enzyme:protein ratio of 1:100 (w/w) and digestion was performed at 37°C for 16 h. The reaction was quenched by acidification to a final concentration of 0.1% TFA and precipitated SDC was removed by centrifugation. Peptides were purified on a SepPak C18 column (Waters, USA) and were eluted in two steps (40% ACN followed by 60% ACN in ddH$_2$O).

1.8.3 Left ventricle High-pH offline fractionation for data dependent acquisition spectral library generation

3 µg peptide from each left ventricle sample was pooled (72 µg total) and fractionated by reverse-phase high pressure liquid chromatography on a Dionex UltiMate 3000 HPLC system (Thermo Scientific, USA). Peptides were separated on a Waters CSH C18 analytical column (1 mm x 150 mm, 130Å, 1.7 µm) in a gradient mix of Buffer A (5 mM ammoniumbicarbonate) and Buffer B (90% ACN, 5 mM ammoniumbicarbonate). The flow rate was 30 µL/min and fractions were collected by 3 min to 11.4 min with 252s/fraction; from 11.4 min to 42 min with 122.4s/fraction; and from 42 min to 46 min with 80s/fraction. Peptides were subsequently concatenated into 20 fractions. All fractions were dried and resuspended in 5% formic acid, 3% ACN. For the plasma library 40 µg of peptides were fractionated by high-pH reverse-phase fractionation as above and collected into 20 fractions without concatenation.

1.8.4 Left ventricle liquid chromatography and mass spectrometry

Peptide were separated by a linear ACN gradient for 60 min in a 15-cm fused-silica emitter packed with reversed-phase ReproSil-Pur C18-AQ 1.9 µm resin (Dr. Maisch GmbH) using a nanoflow Easy-nLC 1200 ultra-high pressure system (Thermo Fisher Scientific). The LC was connected through a nano-electrospray ion source to the mass spectrometer. We used a QExactive HF orbitrap instrument with a scan range between 300 to 1600 m/z, 60,000 resolution and a top12 higher-energy collisional dissociation (HCD) fragmentation method for the data-dependent library experiment. Resolution for HCD spectra was set to 30,000 at *m/z* 200 and an IT of 45 ms. Normalized collision energy (NCE) was set at 28. Precursor ions with single, unassigned, or six and higher charge states were excluded from fragmentation selection. For individual samples we employed a data-independent (DIA) setup with full MS resolutions set to 60,000 at m/z 200 and full MS AGC target was 3E6 with an injection time of 45 ms. Scan range was set to 350–1400 m/z. AGC-target value for fragment spectra was set at 3E6. We used a variable window size with an overlap of 1 Da. Resolution was set to 30,000 and IT to 50 ms. Normalized CE was set at 25 eV. All data were acquired in profile mode using positive polarity with dynamic exclusion set to 30 s to avoid repeated sequencing of the same peptide.

1.8.5 Proteomics data analysis

The data was searched using the Pulsar search engine (Spectronaut version 15.1.210713.50606, Biognosys). The data-dependent and data-independent raw files were used to generate a hybrid library using BSG factory settings. Peptides were searched against the *Mus musculus* Swissprot reference database (downloaded 2017). Search criteria included cysteine carbamidomethylation as a fixed modification as well as N-terminal acetylation and methionine oxidation as variable modifications. The false discovery rate was set to 0.01 for both proteins and peptides, as determined by searching a

reverse decoy database. Cleavage specificity was set as C-terminal to arginine and lysine for trypsin/P, with a maximum number of two missed cleavages and a minimum peptide length of seven amino acids during the database search.

The data was further processed using Perseus software, version 1.6.15.0, filtering using minimum four valid values in each condition group. Sema-3 (sample 434) was removed as an outlier. We did a quantile normalization of the data using an R-script, and a two-sample t-test correcting for multiple hypothesis testing for identifying significant regulated proteins. Data are presented as difference of average (>0.5) and the -log10 Student T test ($p < 0.05$) were used to compare between groups. Enriched protein sets were identified using Gprofiler (e106_eg53_p16_65fcd97, database updated on 18/05/2022)with significant g:SCS Threshold $p < 0.05$. Mass spectrometry data have been deposited to the ProteomeXchange Consortium via the PRIDE partner repository with data set identifier PXD034625.

Extended data sets

Supplemental figures

Supplemental Figure 1. Total plasma ketone bodies

Total ketones bodies measured in the plasma

CTRL, control chow; HFD, high fat diet; ANGII, angiotensin-II; Veh, daily injections with Vehicle; Sema, daily injections with semaglutide; PF, pair feeding. Statistical testing was performed with Kruskal–Wallis test followed by Dunn's test. *$P < 0.05$ is considered significant.

Supplemental Figure 2. Proteomics left ventricle unique pathways for pair feeding

Curated Gene Ontology (GO) Biological Processes list of protein identified in the left ventricle demonstrating distinctive pathways engaged by PF. Circle size reflects gene ratio which is proportion of differentially expressed genes in a pathway divided by all differentially expressed genes that map to GO term. Color coding reflects P-value after g:SCS Threshold ($p < 0.05$).

Supplemental Figure 3. Cell type identification of snRNA seq

Manual cell type annotation of clusters was based on both human and mouse gene markers.

Supplemental Figure 4. UMAP per condition

Uniform manifold approximation and projection (UMAP) plot of processed snRNA-seq data from left ventricle tissue per condition (Ctrl, Vehicle, Sema and PF mice). Coloring indicates manual annotation of cell type clusters generated by unsupervised clustering.

Supplemental Figure 5. Proportion of cells per condition

Proportion of cells per condition (Ctrl, Vehicle, Sema and PF mice)

Supplemental Figure 6. Proportion of cells from each cell type

Proportion of cells from each cell type within each condition (Ctrl, Vehicle, Sema and PF mice)

Supplemental Figure 7. The effect of SoupX

SoupX-cleaned count matrices.

Supplemental Figure 8. Regulated genes in specific endothelial cell types comparing semaglutide treatment against vehicle

Dot plot showing regulated genes in specific endothelial cell types comparing semaglutide treatment against Vehicle. Dot size represents the absolute log2 fold change of a gene, while the color indicates positive (POS, orange) or negative (NEG, blue) regulation of a gene.

Supplemental figures and tables

Supplemental Figure 1 – total plasma ketone bodies

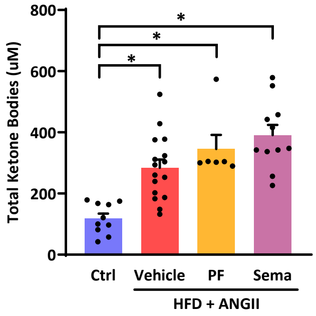

Supplemental Figure 2 Proteomics left ventricle unique pathways pair feeding

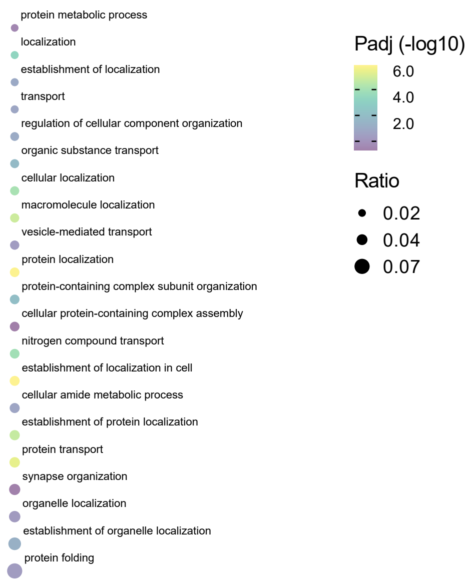

Figure S3 Cell type identification

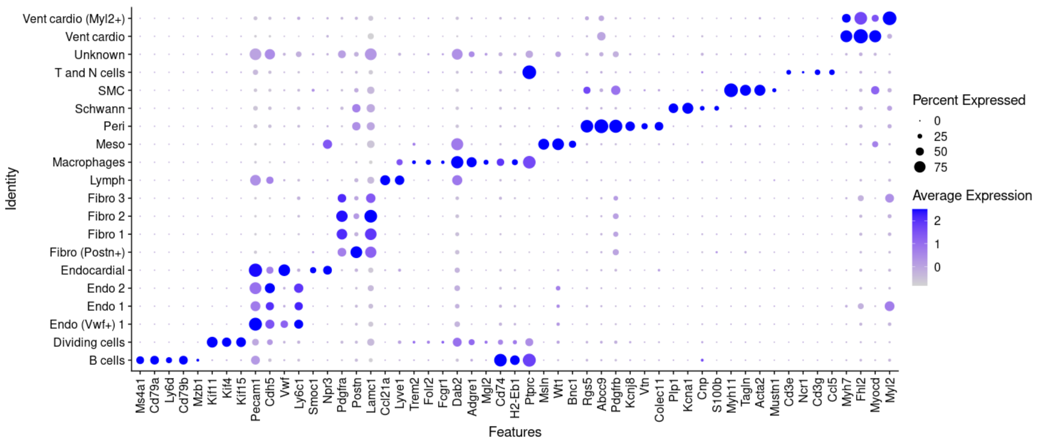

Figure S4 UMAP per condition

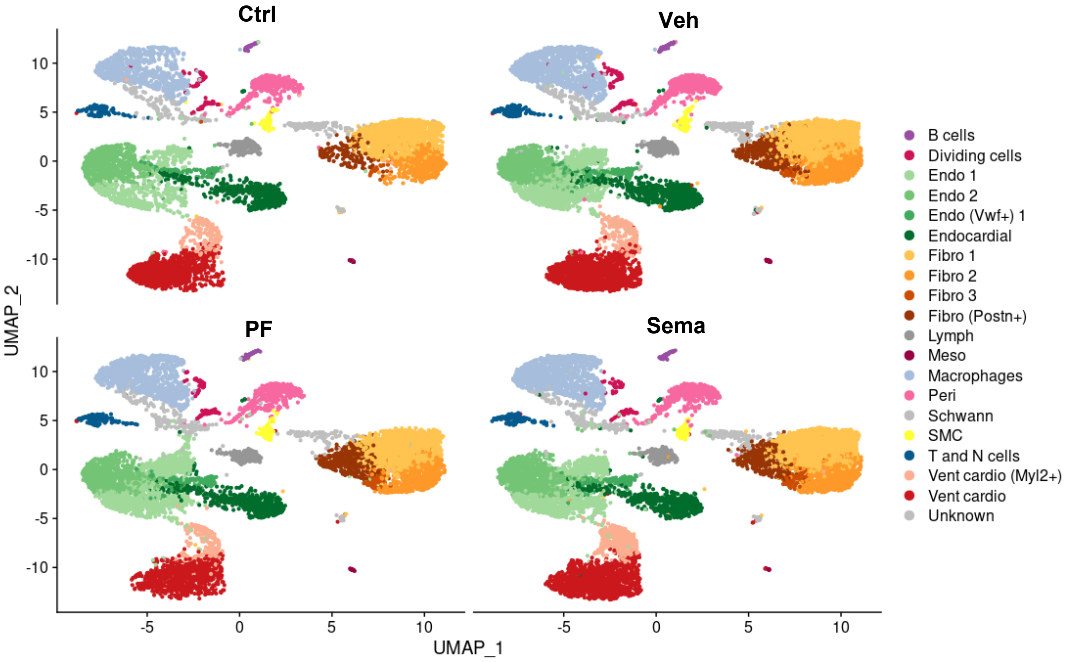

Figure S5 proportion of cells per condition

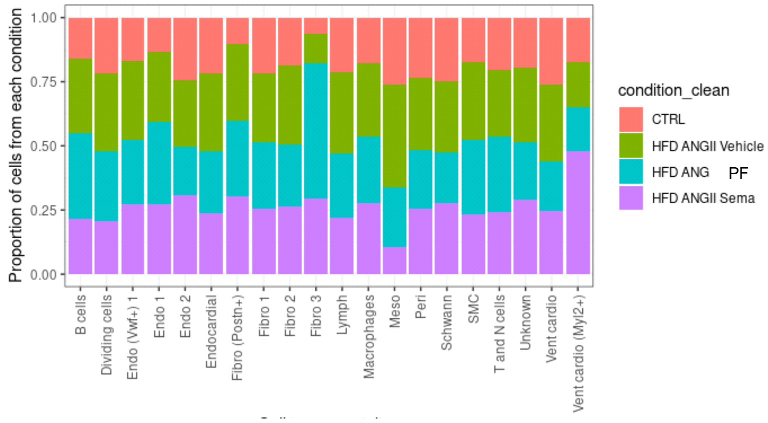

Figure S6 proportion of cells from each cell type

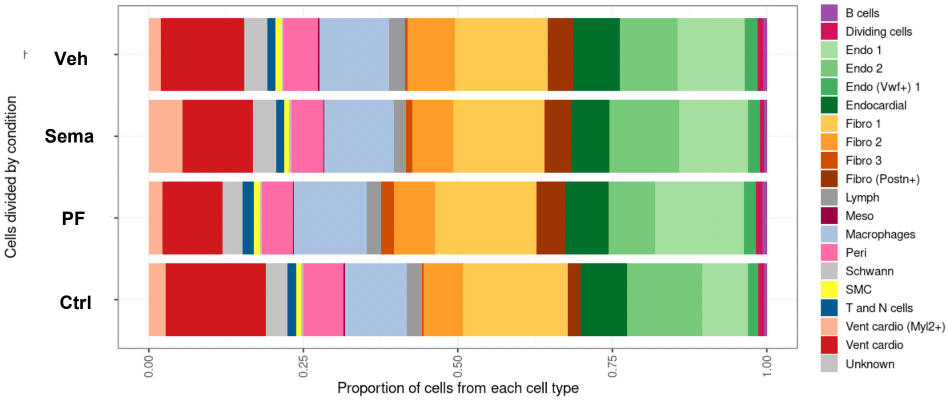

Figure S7 the effect of SoupX

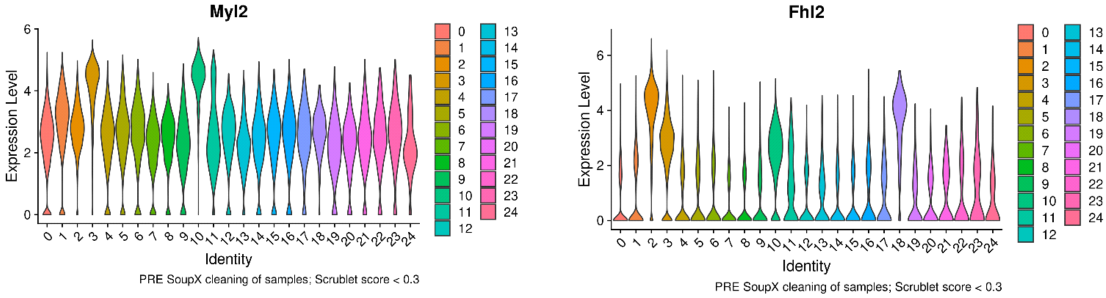

Supplemental Figure 8 Regulated genes in specific endothelial cell types comparing semaglutide treatment against Vehicle

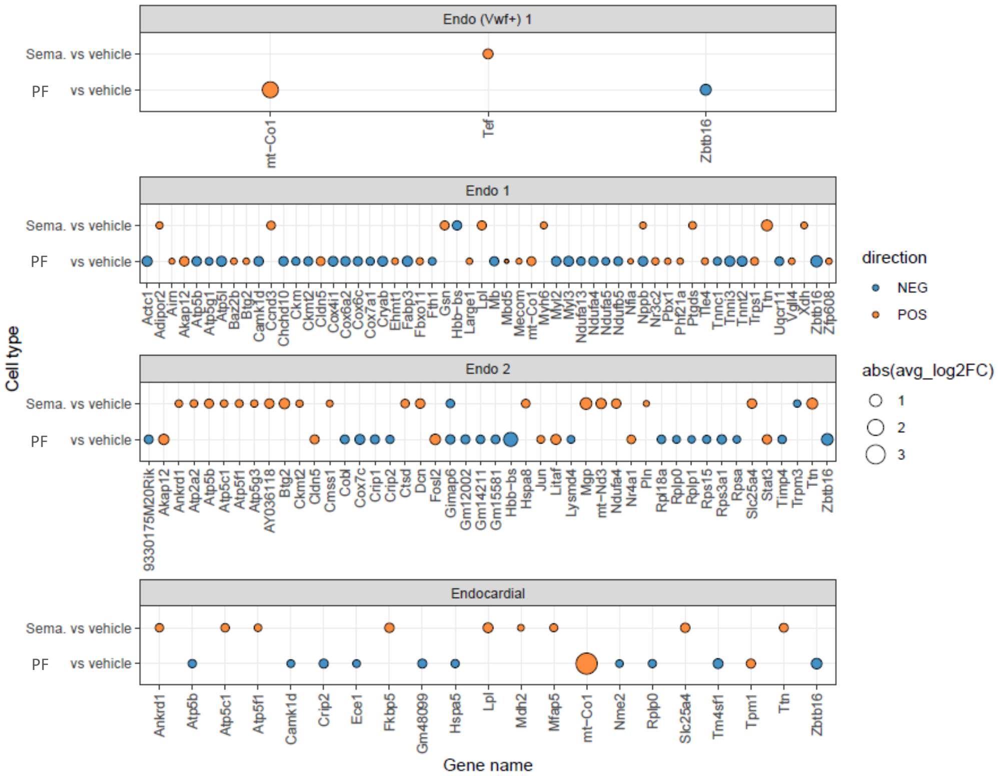

Supplemental tables

Supplemental Table 1. Primer sequences used for qPCR.

Supplemental Table 2. Echocardiography parameters.

LVAW;s, left ventricle anterior wall thickness in systole; LVAW;d, left ventricle anterior wall thickness in diastole LVPW;s, left ventricle posterior wall thickness in systole LVPW;d, left ventricle posterior wall thickness in diastole; GLS, global longitudinal strain; RPLSR, reverse peak longitudinal strain rate.

CTRL, control chow; HFD, high fat diet; ANGII, angiotensin-II; Veh, daily injections with Vehicle; Sema, daily injections with semaglutide; PF, pair feeding. Statistical testing was performed with Kruskal–Wallis test followed by Dunn's test. *P < 0.05 is considered significant.

Supplemental Table 3. LV RNA expression

Gene expression was normalized to 36B4 and presented as fold change to CTRL. Data are presented as means ± standard error of the mean. Statistical testing was performed with Kruskal–Wallis test followed by Mann–Whitney U test. *P < 0.05 compared to CTRL. #P < 0.05 compared to HFD+ANGII Vehicle. ANP, atrial natriuretic peptide; Col1a1, collagen 1a1; Col3a1, collagen 3a1; Gal-3, galectin-3; GDF15, growth derived factor 15; IL-6, interleukin-6; TIMP-1, tissue inhibitor of metalloproteinases 1.

CTRL, control chow; HFD, high fat diet; ANGII, angiotensin-II; Veh, daily injections with Vehicle; Sema, daily injections with semaglutide; PF, pair feeding. Statistical testing was performed with Kruskal–Wallis test followed by Dunn's test. *P < 0.05 is considered significant.

Supplemental Table 4. Pathways and lead edge genes that are differently regulated with semaglutide or pair feeding treatment in endothelial cells or cardiac cells.

Gene ontology, biological processes enrichment analysis of up- or downregulated genes in endothelial cells and cardiac cells comparing semaglutide against Vehicle and pair feeding (PF) against Vehicle.

Supplemental Table 5. Pathways and lead edge genes that are differently regulated with semaglutide or pair feeding treatment in cardiac cells.

Gene ontology, biological processes enrichment analysis of up- or downregulated genes in cardiac cells comparing semaglutide against Vehicle and pair feeding (PF) against Vehicle.

Supplemental Table 6. Pathways and lead edge proteins that are differently regulated with semaglutide or pair feeding treatment in left ventricle tissue

Gene ontology, biological processes enrichment analysis of up- or downregulated proteins in left ventricle tissue comparing semaglutide against Vehicle and pair feeding (PF) against Vehicle.

Supplemental Table 1

Gene	Protein	5' - 3' forward	5' - 3' reverse
Nppa	ANP	ATGGGCTCCTTCTCCATCAC	TCTACCGGCATCTTCTCCTC
TIMP-1	TIMP-1	CTGCTCAGCAAAGAGCTTTC	CTCCAGTTTGCAAGGGATAG
Col1a1	Col1a1	CTTCACCTACAGCACCCTTGTG	CTTGGTGGTTTTGTATTCGATGAC
Col3a1	Col3a1	GCGATTCAAGGCTGAAG	GGGTGCGATATCTATGATGG
Il-6	IL-6	TCCCAACAGACCTGTCTATAC	CAGAATTGCCATTGCACAACTC
Gdf-15	GDF-15	TGACCCAGCTGTCCGGATAC	GTGCACGCGGTAGGCTTC
Rplp0	36B4	AAGCGCGTCCTGGCATTGTC	GCAGCCGCAAATGCAGATGG

Supplemental Table 2

	Ctrl	HFD+ANGII	HFD+ANGII	HFD+ANGII
		**Veh**	**PF**	**Sema**
**Echocardiography**	n = 10	n = 16	n = 8	n = 12
Heart Rate	440.7 ± 13.6	413.1 ± 9.5	451.9 ± 20.6	479.2 ± 15.9 #
Diameter;s	3.0 ± 0.1	2.5 ± 0.1 *	2.8 ± 0.2	2.8 ± 0.1
Diameter;d	4.1 ± 0.1	3.7 ± 0.1 *	4.0 ± 0.1	3.9 ± 0.1
Volume;s	30.4 ± 4.0	23.6 ± 2.5	30.4 ± 4.0	30.1 ± 2.9
Volume;d	76.0 ± 6.5	58.5 ± 4.0	70.5 ± 5.8	65.1 ± 3.6
Stroke Volume	39.5 ± 3.0	35.0 ± 1.9	40.1 ± 2.4	35.0 ± 1.7
Ejection Fraction	52.7 ± 2.8	61.1 ± 2.2	57.9 ± 3.0	54.5 ± 2.6
Fractional Shortening	27.0 ± 1.8	32.4 ± 1.6	30.3 ± 1.5	28.0 ± 1.9
LVAW;s	1.5 ± 0.1	1.8 ± 0.0 *	1.7 ± 0.0 *	1.6 ± 0.1 #
LVAW;d	1.0 ± 0.1	1.3 ± 0.0 *	1.3 ± 0.0 *	1.2 ± 0.1
LVPW;s	1.3 ± 0.1	1.8 ± 0.1 *	1.7 ± 0.1 *	1.5 ± 0.1 #
LVPW;d	1.0 ± 0.0	1.3 ± 0.1 *	1.3 ± 0.1 *	1.1 ± 0.1 #
GLS	-19.0 ± 0.8	-15.9 ± 0.5 *	-16.8 ± 0.9	-20.3 ± 1.1 #
RPLSR	10.4 ± 0.8	8.3 ± 0.6 *	9.0 ± 0.8	11.5 ± 1.0 #

Supplemental Table 3

	Ctrl	HFD+ANGII	HFD+ANGII	HFD+ANGII
		Veh	PF	Sema
**LV RNA expression levels**	n = 10	n = 16	n = 8	n = 12
ANP	1 ± 0.2	2.05 ± 0.2 *	2.05 ± 0.2	1.33 ± 0.2 #
Col1a1	1 ± 0.1	2.72 ± 0.4 *	1.88 ± 0.2	1.37 ± 0.2 #
Col3a1	1 ± 0.1	1.70 ± 0.1 *	1.68 ± 0.3	1.15 ± 0.2 #
TIMP-1	1 ± 0.1	2.88 ± 0.4 *	3.50 ± 0.8	1.88 ± 0.3 #
Gal-3	1 ± 0.1	2.07 ± 0.3 *	1.64 ± 0.2	1.03 ± 0.1 #
IL-6	1 ± 0.3	2.85 ± 0.6 *	3.54 ± 0.9	1.21 ± 0.5 #
GDF-15	1 ± 0.1	2.73 ± 0.3 *	1.23 ± 0.2	1.39 ± 0.3 #

Supplemental Table 4

Endothelial cell pathways upregulated with sema treatment

term name	term id	adjusted p value	negative log10 of adjusted p value	term size	intersection size	ratio	intersections
calcium ion import into sarcoplasmic reticulum	GO:1990036	0.03885753	1.41	7	2	0.286	ATP2A2,PLN
relaxation of cardiac muscle	GO:0055119	0.00104892	2.98	16	3	0.188	TTN,GSN,ATP2A2
response to muscle stretch	GO:0035994	0.00287181	2.54	22	3	0.136	TTN,GSN,ANKRD1
ATP synthesis coupled proton transport	GO:0015986	0.00376882	2.42	24	3	0.125	ATP5B,ATP5G3,ATP5C1
energy coupled proton transport	GO:0015985	0.00376882	2.42	24	3	0.125	ATP5B,ATP5G3,ATP5C1
relaxation of muscle	GO:0090075	9.5001E-05	4.02	35	4	0.114	TTN,GSN,ATP2A2,PLN
cardiac muscle adaptation	GO:0014887	0.00543451	2.26	27	3	0.111	MYH6,NPPB,ATP2A2
regulation of the force of heart contraction	GO:0002026	0.00677897	2.17	29	3	0.103	MYH6,ATP2A2,PLN
striated muscle adaptation	GO:0014888	0.00041313	3.38	50	4	0.080	GSN,MYH6,NPPB,ATP2A2
cardiac muscle hypertrophy	GO:0003300	0.00012308	3.91	100	5	0.050	TTN,MYH6,NPPB,SLC25A4,ATP2A2
striated muscle hypertrophy	GO:0014897	0.00014988	3.82	104	5	0.048	TTN,MYH6,NPPB,SLC25A4,ATP2A2
muscle hypertrophy	GO:0014896	0.00016491	3.78	106	5	0.047	TTN,MYH6,NPPB,SLC25A4,ATP2A2
actin-mediated cell contraction	GO:0070252	0.00575533	2.24	96	4	0.042	GSN,MYH6,ATP2A2,PLN
muscle adaptation	GO:0043500	0.00036142	3.44	124	5	0.040	GSN,MYH6,NPPB,SLC25A4,ATP2A2
cardiac muscle contraction	GO:0060048	0.0004574	3.34	130	5	0.038	TTN,GSN,MYH6,ATP2A2,PLN
proton transmembrane transport	GO:1902600	0.00061563	3.21	138	5	0.036	NDUFA4,ATP5B,ATP5G3,SLC25A4,ATP5C1
actin filament-based movement	GO:0030048	0.0159123	1.80	124	4	0.032	GSN,MYH6,ATP2A2,PLN
striated muscle contraction	GO:0006941	0.00177919	2.75	171	5	0.029	TTN,GSN,MYH6,ATP2A2,PLN
purine ribonucleotide biosynthetic process	GO:0009152	0.04667575	1.33	163	4	0.025	NPPB,ATP5B,ATP5G3,ATP5C1
ATP metabolic process	GO:0046034	0.00058471	3.23	264	6	0.023	MYH6,MT-ND3,ATP5B,ATP5G3,ATP5C1,HSPA8

Endothelial cell pathways upregulated with PF treatment

term name	term id	adjusted p value	negative log10 of adjusted p value	term size	intersection size	ratio	intersections
response to electrical stimulus	GO:0051602	0.00012723	3.90	49	4	0.082	MT-CO1,BTG2,AKAP12,NR4A1
protein alkylation	GO:0008213	0.04657039	1.33	214	4	0.019	MECOM,BTG2,EHMT1,FBXO11
protein methylation	GO:0006479	0.04657039	1.33	214	4	0.019	MECOM,BTG2,EHMT1,FBXO11
negative regulation of RNA metabolic process	GO:0051253	0.00262833	2.58	1489	9	0.006	MECOM,ZFP608,BTG2,VGLL4,EHMT1,TLE4,TRPS1,JUN,STAT3
negative regulation of transcription, DNA-templated	GO:0045892	0.01575461	1.80	1375	8	0.006	MECOM,ZFP608,BTG2,VGLL4,EHMT1,TLE4,TRPS1,JUN
negative regulation of nucleic acid-templated transcription	GO:1903507	0.01592334	1.80	1377	8	0.006	MECOM,ZFP608,BTG2,VGLL4,EHMT1,TLE4,TRPS1,JUN
negative regulation of RNA biosynthetic process	GO:1902679	0.01600828	1.80	1378	8	0.006	MECOM,ZFP608,BTG2,VGLL4,EHMT1,TLE4,TRPS1,JUN
regulation of cell population proliferation	GO:0042127	0.00099769	3.00	1764	10	0.006	MECOM,PBX1,BTG2,VGLL4,CLDN5,JUN,NR4A1,STAT3,FOSL2,TPM1
negative regulation of nucleobase-containing compound metabolic process	GO:0045934	0.00528324	2.28	1620	9	0.006	MECOM,ZFP608,BTG2,VGLL4,EHMT1,TLE4,TRPS1,JUN,STAT3
negative regulation of cellular biosynthetic process	GO:0031327	0.00702294	2.15	1677	9	0.005	MECOM,ZFP608,BTG2,VGLL4,EHMT1,TLE4,TRPS1,JUN,STAT3
negative regulation of biosynthetic process	GO:0009890	0.00864453	2.06	1720	9	0.005	MECOM,ZFP608,BTG2,VGLL4,EHMT1,TLE4,TRPS1,JUN,STAT3
regulation of transcription by RNA polymerase II	GO:0006357	2.1315E-05	4.67	2494	13	0.005	NFIA,MECOM,ZFP608,PBX1,BTG2,EHMT1,TLE4,TRPS1,JUN,NR4A1,STAT3,LITAF,FOSL2
transcription by RNA polymerase II	GO:0006366	2.9241E-05	4.53	2560	13	0.005	NFIA,MECOM,ZFP608,PBX1,BTG2,EHMT1,TLE4,TRPS1,JUN,NR4A1,STAT3,LITAF,FOSL2
response to oxygen-containing compound	GO:1901700	0.00336244	2.47	2014	10	0.005	MBD5,BTG2,CLDN5,AKAP12,JUN,NR4A1,STAT3,LITAF,FOSL2,TPM1
positive regulation of RNA metabolic process	GO:0051254	0.01472863	1.83	1836	9	0.005	NFIA,MECOM,PBX1,BTG2,JUN,NR4A1,STAT3,LITAF,FOSL2
cell population proliferation	GO:0008283	0.00510999	2.29	2109	10	0.005	MECOM,PBX1,BTG2,VGLL4,CLDN5,JUN,NR4A1,STAT3,FOSL2,TPM1
positive regulation of macromolecule biosynthetic process	GO:0010557	0.0203829	1.69	1911	9	0.005	NFIA,MECOM,PBX1,AKAP12,JUN,NR4A1,STAT3,LITAF,FOSL2
tissue development	GO:0009888	0.03533652	1.45	2046	9	0.004	MECOM,PBX1,BTG2,VGLL4,CLDN5,JUN,NR4A1,FOSL2,TPM1
positive regulation of nucleobase-containing compound metabolic process	GO:0045935	0.03575443	1.45	2049	9	0.004	NFIA,MECOM,PBX1,BTG2,JUN,NR4A1,STAT3,LITAF,FOSL2
positive regulation of biosynthetic process	GO:0009891	0.04032752	1.39	2080	9	0.004	NFIA,MECOM,PBX1,AKAP12,JUN,NR4A1,STAT3,LITAF,FOSL2

Endothelial cell pathways downregulated with PF treatment

term name	term id	adjusted p value	negative log10 of adjusted p value	term size	intersection size	ratio	intersections
regulation of muscle filament sliding speed	GO:0032972	0.00598089	2.223234	2	2	1.000	TNNT2,TNNC1
phosphocreatine metabolic process	GO:0006603	0.03580199	1.446093	4	2	0.500	CKMT2,CKM
phosphagen metabolic process	GO:0006599	0.03580199	1.446093	4	2	0.500	CKMT2,CKM
regulation of muscle filament sliding	GO:0032971	0.03580199	1.446093	4	2	0.500	TNNT2,TNNC1
phosphagen biosynthetic process	GO:0042396	0.03580199	1.446093	4	2	0.500	CKMT2,CKM
phosphocreatine biosynthetic process	GO:0046314	0.03580199	1.446093	4	2	0.500	CKMT2,CKM
actin-myosin filament sliding	GO:0033275	0.0017024	2.768938	11	3	0.273	ACTC1,TNNT2,TNNC1
mitochondrial electron transport, cytochrome c to oxygen	GO:0006123	7.6459E-07	6.116572	22	5	0.227	COX4I1,COX6A2,COX6C,COX7A1,COX7C
proton motive force-driven ATP synthesis	GO:0015986	0.02053825	1.687437	24	3	0.125	ATP5L,ATP5B,ATP5G1
ventricular cardiac muscle tissue morphogenesis	GO:0055010	7.9874E-05	4.097596	53	5	0.094	TNNI3,MYL3,TNNT2,MYL2,TNNC1
aerobic electron transport chain	GO:0019646	3.3623E-06	5.473362	64	6	0.094	COX4I1,COX6A2,COX6C,UQCR11,COX7A1,COX7C
cardiac muscle tissue morphogenesis	GO:0055008	5.3393E-06	5.272513	69	6	0.087	TNNI3,ACTC1,MYL3,TNNT2,MYL2,TNNC1
ventricular cardiac muscle tissue development	GO:0003229	0.00019299	3.714462	63	5	0.079	TNNI3,MYL3,TNNT2,MYL2,TNNC1
mitochondrial ATP synthesis coupled electron transport	GO:0042775	1.0445E-05	4.981093	77	6	0.078	COX4I1,COX6A2,COX6C,UQCR11,COX7A1,COX7C
ATP synthesis coupled electron transport	GO:0042773	1.2212E-05	4.913218	79	6	0.076	COX4I1,COX6A2,COX6C,UQCR11,COX7A1,COX7C
muscle tissue morphogenesis	GO:0060415	1.5321E-05	4.814721	82	6	0.073	TNNI3,ACTC1,MYL3,TNNT2,MYL2,TNNC1
muscle organ morphogenesis	GO:0048644	2.6944E-05	4.569534	90	6	0.067	TNNI3,ACTC1,MYL3,TNNT2,MYL2,TNNC1
respiratory electron transport chain	GO:0022904	1.8842E-06	5.724877	108	7	0.065	COX4I1,COX6A2,COX6C,UQCR11,COX7A1,NDUFA5,COX7C
oxidative phosphorylation	GO:0006119	1.1394E-07	6.943311	124	8	0.065	COX4I1,COX6A2,ATP5B,CHCHD10,COX6C,UQCR11,COX7A1,COX7C
cardiac ventricle morphogenesis	GO:0003208	0.00056793	3.245708	78	5	0.064	TNNI3,MYL3,TNNT2,MYL2,TNNC1

Supplemental Table 5

cardiac cell pathways upregulated with sema treatment

term name	term id	adjusted p value	negative log10 of adjusted p value	term size	intersection size	ratio	intersections
actin-myosin filament sliding	GO:0033275	0,00850262	2,07	11	3	0,273	ACTC1,TPM1,MYL6
regulation of amyloid fibril formation	GO:1905906	0,018629	1,73	14	3	0,214	APOE,CRYAB,CLU
mitochondrial electron transport, NADH to ubiquinone	GO:0006120	0,00309329	2,51	28	4	0,143	MT-ND6,NDUFV2,NDUFV1,NDUFS3
tricarboxylic acid cycle	GO:0006099	0,00538059	2,27	32	4	0,125	MDH2,SUCLG1,SDHB,IDH2
response to electrical stimulus	GO:0051602	0,03043094	1,52	49	4	0,082	ACTB,SOD2,NR4A1,BTG2
chaperone-mediated protein folding	GO:0061077	0,0037061	2,43	66	5	0,076	HSPA8,HSPB1,HSPA5,CLU,FKBP5
response to cadmium ion	GO:0046686	0,04828244	1,32	55	4	0,073	GSN,HSPA8,SOD2,MT1
regulation of striated muscle contraction	GO:0006942	0,01717365	1,77	90	5	0,056	CCN2,MYL3,NR4A1,CALM1,TNNI3
negative regulation of intrinsic apoptotic signaling pathway	GO:2001243	0,02733272	1,56	99	5	0,051	HSPB1,SOD2,CLU,VDAC2,NDUFS3
protein folding	GO:0006457	0,00010843	3,96	168	8	0,048	CRYAB,HSPA8,HSPB1,NPPB,HSPA5,HSP90AB1,CLU,FKBP5
negative regulation of protein-containing complex assembly	GO:0031333	0,01660861	1,78	154	6	0,039	GSN,CRYAB,TPM1,HSPA5,CLU,VDAC2
muscle contraction	GO:0006936	6,948E-06	5,16	310	11	0,035	GSN,ACTC1,TPM1,MYL6,CSRP3,MYL2,CCN2,MYL3,NR4A1,CALM1,TNNI3
negative regulation of supramolecular fiber organization	GO:1902904	0,03217248	1,49	173	6	0,035	GSN,APOE,CRYAB,HSPA8,TPM1,CLU
regulation of blood circulation	GO:1903522	0,03427462	1,47	262	7	0,027	TPM1,CSRP3,MYL2,CCN2,MYL3,CALM1,TNNI3
regulation of protein stability	GO:0031647	0,01046017	1,98	308	8	0,026	GSN,CRYAB,HSPA8,CD81,SERF2,LAMP1,HSP90AB1,CLU
response to metal ion	GO:0010038	0,00518292	2,29	376	9	0,024	GSN,FABP4,HSPA8,SOD2,HSPA5,MT1,BSG,CALM1,JUND
response to inorganic substance	GO:0010035	0,00023454	3,63	653	13	0,020	GSN,FABP4,MT-ND6,CRYAB,HSPA8,SOD2,HSPA5,MT1,BSG,NR4A1,CALM1,JUND,PRDX1
negative regulation of programmed cell death	GO:0043069	1,3417E-07	6,87	1053	20	0,019	APOE,MECOM,CRYAB,HSPA8,ACTC1,CAMK1D,SLC25A4,HSPB1,SOD2,HSPA5,HSP90AB1,MT1,DUSP1,NR4A1,HIGD1A,CLU,BTG2,YBX1,VDAC2,NDUFS3
mitochondrion organization	GO:0007005	0,01378171	1,86	540	10	0,019	DCN,CFH,MT-ND6,SLC25A4,SOD2,NDUFAB1,COX7A2L,HIGD1A,VDAC2,NDUFS3

cardiac cell pathways upregulated with PF treatment

term name	term id	adjusted p value	negative log10 of adjusted p value	term size	intersection size	ratio	intersections
negative regulation of mitochondrial membrane permeability involved in apoptotic process	GO:1902109	0,02894086	1,54	4	2	0,500	ACAA2,SLC25A4
mitochondrial electron transport, cytochrome c to oxygen	GO:0006123	1,82E-09	8,74	22	6	0,273	MT-CO1,COX7C,COX6C,COX7A2,COX5A,COX4I1
cardiac myofibril assembly	GO:0055003	0,00843747	2,07	20	3	0,150	CSRP3,ACTC1,MYL2
regulation of the force of heart contraction	GO:0002026	0,02676745	1,57	29	3	0,103	CSRP3,MYL3,MYL2
ventricular cardiac muscle tissue development	GO:0003229	0,00639031	2,19	63	4	0,063	TNNI3,MYL3,TPM1,MYL2
myofibril assembly	GO:0030239	0,00770434	2,11	66	4	0,061	CSRP3,ACTC1,TPM1,MYL2
striated muscle cell development	GO:0055002	0,00818376	2,09	67	4	0,060	CSRP3,ACTC1,TPM1,MYL2
cardiac ventricle morphogenesis	GO:0003208	0,01503195	1,82	78	4	0,051	TNNI3,MYL3,TPM1,MYL2
cardiac muscle cell development	GO:0055013	0,03426574	1,47	96	4	0,042	CSRP3,SLC25A4,ACTC1,MYL2
nucleoside triphosphate metabolic process	GO:0009141	0,00320935	2,49	123	5	0,041	MT-ATP8,ATP5C1,ATP5L,ATP5B,ATP5G3
cardiac cell development	GO:0055006	0,0435223	1,36	102	4	0,039	CSRP3,SLC25A4,ACTC1,MYL2
response to reactive oxygen species	GO:0000302	0,00246841	2,61	215	6	0,028	PRDX1,SOD2,HSPA8,MB,TPM1,CRYAB
muscle cell development	GO:0055001	0,04047652	1,39	207	5	0,024	CSRP3,SLC25A4,ACTC1,TPM1,MYL2
ribonucleotide metabolic process	GO:0009259	0,00037994	3,42	390	8	0,021	SUCLG1,MT-ATP8,ATP5C1,ATP5L,MPC2,ACAA2,ATP5B,ATP5G3
purine nucleotide metabolic process	GO:0006163	0,00043493	3,36	397	8	0,020	SUCLG1,MT-ATP8,ATP5C1,ATP5L,MPC2,ACAA2,ATP5B,ATP5G3
ribose phosphate metabolic process	GO:0019693	0,00046052	3,34	400	8	0,020	SUCLG1,MT-ATP8,ATP5C1,ATP5L,MPC2,ACAA2,ATP5B,ATP5G3
purine-containing compound metabolic process	GO:0072521	0,00067855	3,17	421	8	0,019	SUCLG1,MT-ATP8,ATP5C1,ATP5L,MPC2,ACAA2,ATP5B,ATP5G3
response to oxidative stress	GO:0006979	0,00094684	3,02	440	8	0,018	MT-CO1,PRDX1,SOD2,HSPA8,MB,HSPB1,TPM1,CRYAB
organophosphate biosynthetic process	GO:0090407	0,00303157	2,52	514	8	0,016	MT-ATP8,ATP5C1,ATP5L,MPC2,ATP5B,ATP5G3,CKM,FABP3

Supplemental Table 6

Left ventricle pathways enriched with semaglutide treatment

term name	term id	adjusted p value	negative log10 of adjusted p value	term size	intersection size	ratio	intersections
purine deoxyribonucleotide metabolic process	GO:0009151	3.31E-02	1.48	22	3	0.1364	GDA,NUDT16,NT5C2
transcytosis	GO:0045056	3.31E-02	1.48	22	3	0.1364	RAB11B,CLTC,USO1
positive regulation of actin filament bundle assembly	GO:0032233	1.45E-02	1.84	68	5	0.0735	SYNPO2L,SYNPO,PPM1F,SYNPO2,LIMCH1
positive regulation of supramolecular fiber organization	GO:1902905	1.34E-03	2.87	209	11	0.0526	HSPA1A,SYNPO2L,LMOD2,SYNPO,CDC42EP4,PPM1F,GRB2,GDA,SLAIN2,SYNPO2,LIMCH1
positive regulation of cytoskeleton organization	GO:0051495	1.58E-03	2.80	228	11	0.0482	HSPA1A,SYNPO2L,LMOD2,SYNPO,CDC42EP4,PPM1F,GRB2,GDA,SLAIN2,SYNPO2,LIMCH1
positive regulation of protein polymerization	GO:0032273	2.99E-02	1.53	132	6	0.0455	HSPA1A,LMOD2,CDC42EP4,GRB2,GDA,SLAIN2
actin filament bundle assembly	GO:0051017	1.98E-02	1.70	166	7	0.0422	AIF1,SYNPO2L,PDLIM1,SYNPO,PPM1F,SYNPO2,LIMCH1
actin filament bundle organization	GO:0061572	2.06E-02	1.69	169	7	0.0414	AIF1,SYNPO2L,PDLIM1,SYNPO,PPM1F,SYNPO2,LIMCH1
actomyosin structure organization	GO:0031032	1.40E-02	1.85	200	8	0.0400	NRAP,SYNPO2L,LMOD2,PDLIM1,SYNPO,PPM1F,CNN3,LIMCH1
striated muscle cell differentiation	GO:0051146	1.12E-02	1.95	298	10	0.0336	NRAP,SYNPO2L,LMOD2,EHD1,ALPK2,SORBS2,RBM38,CDH2,AKAP6,PDLIM5
heterocycle catabolic process	GO:0046700	9.28E-03	2.03	410	12	0.0293	LSM6,THRAP3,HSPA1A,HMOX2,ETF1,RBM38,SSB,GDA,NUDT16,GRSF1,QKI,NT5C2
cellular nitrogen compound catabolic process	GO:0044270	9.28E-03	2.03	411	12	0.0292	LSM6,THRAP3,HSPA1A,HMOX2,ETF1,RBM38,SSB,GDA,NUDT16,GRSF1,QKI,NT5C2
nucleobase-containing compound catabolic process	GO:0034655	1.40E-02	1.85	381	11	0.0289	LSM6,THRAP3,HSPA1A,ETF1,RBM38,SSB,GDA,NUDT16,GRSF1,QKI,NT5C2
aromatic compound catabolic process	GO:0019439	1.28E-02	1.89	432	12	0.0278	LSM6,THRAP3,HSPA1A,HMOX2,ETF1,RBM38,SSB,GDA,NUDT16,GRSF1,QKI,NT5C2
actin filament organization	GO:0007015	1.39E-02	1.86	438	12	0.0274	AIF1,NRAP,SYNPO2L,LMOD2,PDLIM1,SORBS2,SYNPO,CDC42EP4,PPM1F,GRB2,SYNPO2,LIMCH1
organic cyclic compound catabolic process	GO:1901361	1.54E-02	1.81	458	12	0.0262	LSM6,THRAP3,HSPA1A,HMOX2,ETF1,RBM38,SSB,GDA,NUDT16,GRSF1,QKI,NT5C2
positive regulation of cellular component biogenesis	GO:0044089	9.28E-03	2.03	536	14	0.0261	HSPA1A,SYNPO2L,LMOD2,SYNPO,CDC42EP4,PPM1F,TSG101,GRB2,GDA,SLAIN2,SYNPO2,HGS,LIMCH1,PSMC2
actin filament-based process	GO:0030029	7.06E-03	2.15	792	18	0.0227	AIF1,NRAP,EHBP1,SYNPO2L,LMOD2,PDLIM1,SORBS2,SYNPO,CDC42EP4,ARHGEF7,PPM1F,DSG2,GRB2,AKAP6,SYNPO2,PDLIM5,CNN3,LIMCH1
actin cytoskeleton organization	GO:0030036	1.09E-02	1.96	705	16	0.0227	AIF1,NRAP,EHBP1,SYNPO2L,LMOD2,PDLIM1,SORBS2,SYNPO,CDC42EP4,ARHGEF7,PPM1F,GRB2,SYNPO2,PDLIM5,CNN3,LIMCH1
mRNA metabolic process	GO:0016071	2.87E-02	1.54	662	14	0.0211	LSM6,THRAP3,RNMT,HSPA1A,RBM17,PSPC1,HNRNPA3,ETF1,RBM38,SSB,NUDT16,GRSF1,QKI,HNRNPK
cellular macromolecule catabolic process	GO:0044265	9.28E-03	2.03	1085	21	0.0194	LSM6,THRAP3,TMF1,PSMB3,HSPA1A,USP9X,VPS36,UBE2R2,ETF1,RBM38,DAB2,TSG101,SSB,NUDT16,BAG6,UBE2L3,GRSF1,PCBP2,RNF114,QKI,PSMC2
cellular catabolic process	GO:0044248	5.34E-03	2.27	2059	33	0.0160	LSM6,THRAP3,TMF1,PSMB3,HSPA1A,USP9X,VPS36,STBD1,UBE2R2,HMOX2,ETF1,RBM38,ACBD5,DAB2,TSG101,MAP1LC3A,SSB,GDA,MCL1,NUDT16,SCARB2,BAG6,UBE2L3,GRSF1,GOT1,CLTC,SYNPO2,PCBP2,EHHADH,RNF114,QKI,NT5C2,PSMC2

Left ventricle pathways enriched with pair feeding treatment

term name	term id	adjusted p value	negative log10 of adjusted p value	term size	intersection size	ratio	intersections
establishment of localization in cell	GO:0051649	1.48E-08	7.83	2031	65	0.0320	DPY30,A0A0N4SW73,A0A1W2P7K6,A0A140LHH8,DAB2,CASK,ARFGAP3,CEP290,CHMP5,STXBP1,PDCD6,SVIP,UBE2G2,REPS1,NUPL1,SCN5A,ABCE1,HOOK3,CHMP1A,NAPA,NUP188,DLG1,SCARB2,CACNB2,TMED2,HRAS,RAB11B,PEX19,TXNIP,CALR,STBD1,SNAPIN,NUMA1,CHMP4C,GPIHBP1,NUP62,TSG101,CSE1L,ARFRP1,TMED9,TOM1L2,COPB1,MAP4K4,HNRNPA2B1,SNX5,TMEM30A,ZFAND1,BOD1,DTNBP1,SEC24B,BIN1,PEX5,TOMM34,MAP4,CLTA,ACTN4,TOMM5,IPO7,SAMM50,UBE2O,Q8BHE8,SUN1,TMED5,NACA,GRPEL1
protein localization	GO:0008104	1.64E-08	7.79	2452	73	0.0298	A0A0N4SW73,A0A1W2P7K6,TULP3,DAB2,CASK,ARFGAP3,CEP290,NUP43,CHMP5,STXBP1,APOE,PDCD6,SVIP,UBE2G2,PPFIA1,NUPL1,ANKRD1,HOOK3,CHMP1A,NPNT,NAPA,NUP188,DLG1,SCARB2,CACNB2,TMED2,HRAS,RAB11B,PEX19,TXNIP,CALR,HNRNPK,CDK5RAP3,SNAPIN,NUMA1,CHMP4C,GPIHBP1,NUP62,TSG101,CSE1L,ARFRP1,GLRX,TTN,TMED9,TOM1L2,COPB1,MAP4K4,PPM1F,CLIC5,SNX5,TMEM30A,ZFAND1,BOD1,GOLGB1,SEC24B,PEX5,TOMM34,NEDD8,CLTA,ACTN4,HOMER1,PIN1,TOMM5,IPO7,AKAP2,SAMM50,Q8BHE8,BAG1,SUN1,TMED5,NACA,GRPEL1,TPP1
protein transport	GO:0015031	4.15E-08	7.38	1535	54	0.0352	A0A0N4SW73,A0A1W2P7K6,CASK,ARFGAP3,CEP290,NUP43,CHMP5,STXBP1,APOE,PDCD6,SVIP,UBE2G2,NUPL1,ANKRD1,HOOK3,CHMP1A,NAPA,NUP188,SCARB2,TMED2,HRAS,RAB11B,PEX19,TXNIP,CALR,SNAPIN,CHMP4C,GPIHBP1,NUP62,TSG101,CSE1L,ARFRP1,GLRX,TTN,TMED9,TOM1L2,COPB1,MAP4K4,PPM1F,SNX5,TMEM30A,ZFAND1,SEC24B,PEX5,TOMM34,CLTA,ACTN4,TOMM5,IPO7,SAMM50,Q8BHE8,TMED5,NACA,GRPEL1
macromolecule localization	GO:0033036	1.37E-07	6.86	2897	79	0.0273	HNRNPA3,A0A0N4SW73,A0A1W2P7K6,QKI,TULP3,DAB2,CASK,ARFGAP3,CEP290,NUP43,CHMP5,STXBP1,APOE,PDCD6,SVIP,UBE2G2,PPFIA1,NUPL1,ANKRD1,HOOK3,CHMP1A,NPNT,APOC3,NAPA,NUP188,DLG1,SCARB2,CACNB2,TMED2,HRAS,RAB11B,PEX19,TXNIP,CALR,HNRNPK,CDK5RAP3,SNAPIN,NUMA1,CHMP4C,GPIHBP1,NUP62,TSG101,CSE1L,ARFRP1,GLRX,TTN,TMED9,TOM1L2,COPB1,MAP4K4,HNRNPA2B1,PPM1F,CLIC5,SNX5,TMEM30A,ZFAND1,BOD1,GOLGB1,SEC24B,PEX5,TOMM34,NEDD8,CLTA,ACTN4,LIMA1,HOMER1,PIN1,TOMM5,IPO7,AKAP2,SAMM50,Q8BHE8,BAG1,SUN1,TMED5,NACA,GRPEL1,RETN,TPP1
establishment of protein localization	GO:0045184	1.89E-07	6.72	1647	55	0.0334	A0A0N4SW73,A0A1W2P7K6,CASK,ARFGAP3,CEP290,NUP43,CHMP5,STXBP1,APOE,PDCD6,SVIP,UBE2G2,NUPL1,ANKRD1,HOOK3,CHMP1A,NPNT,NAPA,NUP188,SCARB2,TMED2,HRAS,RAB11B,PEX19,TXNIP,CALR,SNAPIN,CHMP4C,GPIHBP1,NUP62,TSG101,CSE1L,ARFRP1,GLRX,TTN,TMED9,TOM1L2,COPB1,MAP4K4,PPM1F,SNX5,TMEM30A,ZFAND1,SEC24B,PEX5,TOMM34,CLTA,ACTN4,TOMM5,IPO7,SAMM50,Q8BHE8,TMED5,NACA,GRPEL1
cellular localization	GO:0051641	7.98E-07	6.10	2777	75	0.0270	DPY30,A0A0N4SW73,A0A1W2P7K6,A0A140LHH8,TULP3,DAB2,CASK,ARFGAP3,CEP290,CHMP5,STXBP1,APOE,PDCD6,SVIP,UBE2G2,REPS1,PPFIA1,NUPL1,SCN5A,ABCE1,HOOK3,CHMP1A,NAPA,NUP188,DLG1,SCARB2,CACNB2,TMED2,HRAS,RAB11B,PEX19,TXNIP,CALR,HNRNPK,CDK5RAP3,STBD1,SNAPIN,NUMA1,CHMP4C,GPIHBP1,NUP62,TSG101,CSE1L,ARFRP1,TMED9,TOM1L2,COPB1,MAP4K4,HNRNPA2B1,PPM1F,SNX5,TMEM30A,ZFAND1,BOD1,DTNBP1,GOLGB1,SEC24B,BIN1,PEX5,TOMM34,MAP4,CLTA,ACTN4,PIN1,TOMM5,IPO7,SAMM50,UBE2O,Q8BHE8,BAG1,SUN1,TMED5,NACA,GRPEL1,TPP1
nitrogen compound transport	GO:0071705	1.18E-06	5.93	1986	60	0.0302	HNRNPA3,A0A0N4SW73,A0A1W2P7K6,QKI,CASK,ARFGAP3,CEP290,NUP43,CHMP5,STXBP1,APOE,PDCD6,SVIP,UBE2G2,NUPL1,ANKRD1,HOOK3,CHMP1A,NAPA,S100A8,NUP188,SCARB2,TMED2,HRAS,RAB11B,PEX19,TXNIP,CALR,SNAPIN,CHMP4C,GPIHBP1,NUP62,TSG101,CSE1L,ARFRP1,GLRX,TTN,TMED9,TOM1L2,COPB1,MAP4K4,HNRNPA2B1,PPM1F,SNX5,TMEM30A,ZFAND1,DTNBP1,SEC24B,ARHGEF7,PEX5,TOMM34,CLTA,ACTN4,TOMM5,IPO7,SAMM50,Q8BHE8,TMED5,NACA,GRPEL1
localization	GO:0051179	4.77E-06	5.32	6168	127	0.0206	DPY30,HNRNPA3,A0A0N4SW73,A0A1W2P7K6,QKI,A0A140LHH8,TULP3,DAB2,CASK,ARFGAP3,CEP290,PKN1,HBB-B1,NUP43,FSCN1,LGALS3BP,CHMP5,STXBP1,APOE,PDCD6,DYNC1LI2,SVIP,UBE2G2,REPS1,PPFIA1,CD163,NUPL1,ANKRD1,SCN5A,ABCE1,SASH1,HOOK3,CHMP1A,WNK1,NPNT,APOC3,NAPA,JCAD,S100A8,EFNB1,NUP188,COMMD9,DLG1,SCARB2,CACNB2,CLINT1,TMED2,HRAS,RAB11B,PEX19,TXNIP,CALR,LIMCH1,HNRNPK,CDK5RAP3,STBD1,PDLIM1,SNAPIN,FADD,NUMA1,CHMP4C,GPIHBP1,ATP6V1G1,NUP62,RRAS,ECM1,CD44,TSG101,CSE1L,PALLD,ARFRP1,GLRX,LAMA4,TTN,IFITM3,TMED9,TOM1L2,COPB1,LAMB1,MAP4K4,LGMN,HNRNPA2B1,PPM1F,S100A11,DSG2,LRPAP1,CLIC5,SNX5,TMEM30A,ZFAND1,DNAJA4,BOD1,PHACTR4,DTNBP1,GOLGB1,SEC24B,ARHGEF7,BIN1,MCAM,PEX5,TOMM34,NEDD8,MICAL3,MAP4,CLTA,ACTN4,CLIC1,LIMA1,HOMER1,COX17,PIN1,TOMM5,IPO7,AKAP2,BNIP3,COX7A2,SAMM50,UBE2O,Q8BHE8,BAG1,SUN1,TMED5,NACA,GRPEL1,RETN,FNBP1L,TPP1
protein-containing complex subunit organization	GO:0043933	8.32E-05	4.08	1636	49	0.0300	TMOD2,RPL3,VASP,FSCN1,STXBP1,APOE,PDCD6,SVIP,CKAP5,RPS28,ABCE1,MGP,TAF10,NAPA,EIF3M,DLG1,HPRT1,SNRPC,CALR,NAP1L4,SNAPIN,NUMA1,CAPG,GPIHBP1,OTUD6B,PALLD,TTN,A0A0R4J0T0,ZFAND1,NDUFB9,RPS27L,ARHGEF7,BIN1,PEX5,SPTAN1,MICAL3,CLTA,SET,LIMA1,HOMER1,COX17,BNIP3,ETF1,EIF4G1,COX7A2,SAMM50,FARSB,TBCA,STUB1
organic substance transport	GO:0071702	1.21E-04	3.92	2416	63	0.0261	HNRNPA3,A0A0N4SW73,A0A1W2P7K6,QKI,CASK,ARFGAP3,CEP290,NUP43,CHMP5,STXBP1,APOE,PDCD6,SVIP,UBE2G2,NUPL1,ANKRD1,HOOK3,CHMP1A,APOC3,NAPA,S100A8,NUP188,SCARB2,TMED2,HRAS,RAB11B,PEX19,TXNIP,CALR,SNAPIN,CHMP4C,GPIHBP1,NUP62,TSG101,CSE1L,ARFRP1,GLRX,TTN,TMED9,TOM1L2,COPB1,MAP4K4,HNRNPA2B1,PPM1F,SNX5,TMEM30A,ZFAND1,DTNBP1,SEC24B,ARHGEF7,PEX5,TOMM34,CLTA,ACTN4,LIMA1,TOMM5,IPO7,SAMM50,Q8BHE8,TMED5,NACA,GRPEL1,RETN
establishment of organelle localization	GO:0051656	4.52E-04	3.35	386	20	0.0518	A0A140LHH8,ARFGAP3,CHMP5,STXBP1,PDCD6,ABCE1,HOOK3,CHMP1A,DLG1,RAB11B,SNAPIN,NUMA1,CHMP4C,NUP62,TMED9,BOD1,DTNBP1,MAP4,ACTN4,SUN1
regulation of cellular component organization	GO:0051128	7.74E-04	3.11	2428	61	0.0251	A0A1W2P7K6,QKI,TMOD2,DAB2,CASK,VASP,FSCN1,CHMP5,STXBP1,APOE,SVIP,CKAP5,PPFIA1,PFDN4,CCDC85B,ANKRD1,CHMP1A,NAPA,DLG1,SYNPO,SCARB2,RBBP7,HRAS,CALR,LIMCH1,HNRNPK,SNAPIN,NUMA1,CAPG,CHMP4C,GPIHBP1,NUP62,CD44,TSG101,PALLD,TMED9,TOM1L2,MAP4K4,TUBB5,HNRNPA2B1,PPM1F,LRPAP1,TMEM30A,DNAJA4,DTNBP1,ARHGEF7,BIN1,PEX5,SYNPO2L,SPTAN1,MAP4,ACTN4,LIMA1,HOMER1,PFDN1,PIN1,TUBA1A,BNIP3,ETF1,EIF4G1,STUB1
establishment of localization	GO:0051234	8.96E-04	3.05	4658	97	0.0208	DPY30,HNRNPA3,A0A0N4SW73,A0A1W2P7K6,QKI,A0A140LHH8,DAB2,CASK,ARFGAP3,CEP290,HBB-B1,NUP43,LGALS3BP,CHMP5,STXBP1,APOE,PDCD6,SVIP,UBE2G2,REPS1,CD163,NUPL1,ANKRD1,SCN5A,ABCE1,HOOK3,CHMP1A,WNK1,NPNT,APOC3,NAPA,S100A8,NUP188,COMMD9,DLG1,SCARB2,CACNB2,CLINT1,TMED2,HRAS,RAB11B,PEX19,TXNIP,CALR,HNRNPK,STBD1,SNAPIN,NUMA1,CHMP4C,GPIHBP1,ATP6V1G1,NUP62,TSG101,CSE1L,ARFRP1,GLRX,TTN,IFITM3,TMED9,TOM1L2,COPB1,MAP4K4,HNRNPA2B1,PPM1F,LRPAP1,CLIC5,SNX5,TMEM30A,ZFAND1,BOD1,DTNBP1,SEC24B,ARHGEF7,BIN1,PEX5,TOMM34,MICAL3,MAP4,CLTA,ACTN4,CLIC1,LIMA1,HOMER1,COX17,TOMM5,IPO7,BNIP3,COX7A2,SAMM50,UBE2O,Q8BHE8,SUN1,TMED5,NACA,GRPEL1,RETN,FNBP1L
transport	GO:0006810	1.35E-03	2.87	4502	94	0.0209	DPY30,HNRNPA3,A0A0N4SW73,A0A1W2P7K6,QKI,A0A140LHH8,DAB2,CASK,ARFGAP3,CEP290,HBB-B1,NUP43,LGALS3BP,CHMP5,STXBP1,APOE,PDCD6,SVIP,UBE2G2,REPS1,CD163,NUPL1,ANKRD1,SCN5A,ABCE1,HOOK3,CHMP1A,WNK1,APOC3,NAPA,S100A8,NUP188,COMMD9,DLG1,SCARB2,CACNB2,CLINT1,TMED2,HRAS,RAB11B,PEX19,TXNIP,CALR,HNRNPK,STBD1,SNAPIN,NUMA1,CHMP4C,GPIHBP1,ATP6V1G1,NUP62,TSG101,CSE1L,ARFRP1,GLRX,TTN,IFITM3,TMED9,TOM1L2,COPB1,MAP4K4,HNRNPA2B1,PPM1F,LRPAP1,CLIC5,SNX5,TMEM30A,ZFAND1,DTNBP1,SEC24B,ARHGEF7,BIN1,PEX5,TOMM34,MICAL3,CLTA,ACTN4,CLIC1,LIMA1,HOMER1,COX17,TOMM5,IPO7,BNIP3,COX7A2,SAMM50,UBE2O,Q8BHE8,SUN1,TMED5,NACA,GRPEL1,RETN,FNBP1L
cellular amide metabolic process	GO:0043603	1.51E-03	2.82	1077	35	0.0325	QKI,RPL26,RPL3,APOE,EIF2B3,RPS28,ABCE1,PGAM1,EIF3M,TMED2,MRPS6,MRPL39,CALR,PURB,MRPL55,YARS,OTUD6B,SYNCRIP,MRPL17,CLIC5,RPS5,RPS27L,BIN1,AARS,MRPL27,ZC3H15,MRPS18C,CLIC1,PIN1,TSFM,ETF1,EIF4G1,RPL34,FARSB,TPP1
vesicle-mediated transport	GO:0016192	3.48E-03	2.46	1582	44	0.0278	DPY30,A0A0N4SW73,A0A1W2P7K6,DAB2,CASK,ARFGAP3,LGALS3BP,CHMP5,STXBP1,APOE,PDCD6,REPS1,CD163,HOOK3,CHMP1A,NAPA,SCARB2,CACNB2,CLINT1,TMED2,HRAS,RAB11B,CALR,HNRNPK,SNAPIN,CHMP4C,GPIHBP1,TSG101,ARFRP1,GLRX,IFITM3,TMED9,COPB1,LRPAP1,SNX5,DTNBP1,SEC24B,BIN1,MICAL3,CLTA,ACTN4,UBE2O,TMED5,FNBP1L
protein folding	GO:0006457	3.51E-03	2.45	162	12	0.0741	FKBP9,PFDN4,NUDCD2,PEX19,CALR,DNAJB4,DNAJA4,PFDN1,TBCA,BAG1,STUB1,GRPEL1
organelle localization	GO:0051640	4.39E-03	2.36	528	22	0.0417	A0A140LHH8,ARFGAP3,CEP290,CHMP5,STXBP1,PDCD6,ABCE1,HOOK3,CHMP1A,DLG1,RAB11B,SNAPIN,NUMA1,CHMP4C,NUP62,TMED9,BOD1,DTNBP1,BIN1,MAP4,ACTN4,SUN1
protein metabolic process	GO:0019538	2.33E-02	1.63	5474	104	0.0190	DPY30,UBE2R2,QKI,FRY,DAB2,RPL26,CASK,NFS1,PKN1,PSMD12,RPL3,ADAMTSL4,APOE,PDCD6,SVIP,NAA30,UBE2G2,FKBP9,EIF2B3,RPS28,PFDN4,ABCE1,SASH1,USP10,WNK1,APOC3,TAF10,S100A8,EIF3M,TLR3,DLG1,UBE2G1,TMED2,HRAS,UBE2L3,MRPS6,PSMA5,MRPL39,CALR,PURB,LIMCH1,RNF114,CDK5RAP3,MRPL55,YARS,SNAPIN,ITIH3,FADD,LIAS,CHMP4C,NUP62,OTUD6B,SYNCRIP,PCBP2,ECM1,FAM98B,CD44,TSG101,CCNL1,ITM2B,TTN,F10,PPTC7,A0A0R4J0T0,MAP4K4,LGMN,ART3,MRPL17,PPM1F,PSMB1,TRIM63,SNX5,RPS5,PRSS1,BOD1,PHACTR4,F9,DTNBP1,RPS27L,DOHH,BIN1,AARS,MRPL27,CASP7,ZC3H15,MRPS18C,PRDM16,NEDD8,PRKRA,PFDN1,PIN1,TSFM,BNIP3,ETF1,EIF4G1,RPL34,UBE2O,FARSB,MKNK1,BAG1,STUB1,ALPK2,NUDT16,TPP1
synapse organization	GO:0050808	3.84E-02	1.42	474	19	0.0401	CASK,APOE,PPFIA1,EFNB1,DLG1,SYNPO,CACNB2,HNRNPK,SNAPIN,PALLD,LGMN,TUBB5,PPFIBP1,DTNBP1,ARHGEF7,HOMER1,PIN1,TUBA1A,EIF4G1
cellular protein-containing complex assembly	GO:0034622	4.38E-02	1.36	996	30	0.0301	TMOD2,RPL3,VASP,STXBP1,CKAP5,RPS28,TAF10,NAPA,EIF3M,DLG1,SNRPC,CALR,NAP1L4,NUMA1,CAPG,OTUD6B,TTN,NDUFB9,RPS27L,ARHGEF7,BIN1,SPTAN1,CLTA,SET,COX17,EIF4G1,COX7A2,SAMM50,TBCA,STUB1
